# Transcriptomic Profiling of the Liver Sinusoidal Endothelium during Cirrhosis Reveals Stage-Specific Secretory Signature

**DOI:** 10.3390/cancers13112688

**Published:** 2021-05-29

**Authors:** Nicolò Manicardi, Anabel Fernández-Iglesias, Laia Abad-Jordà, Felix Royo, Mikel Azkargorta, Martí Ortega-Ribera, David Sanfeliu-Redondo, Ana Martínez-Alcocer, Felix Elortza, Amelia J. Hessheimer, Constantino Fondevila, Juan José Lozano, Juan Carlos García-Pagán, Jaime Bosch, Francisco Javier Cubero, Agustín Albillos, Javier Vaquero, Juan M. Falcón-Pérez, Jordi Gracia-Sancho

**Affiliations:** 1 Liver Vascular Biology Research Group, Barcelona Hepatic Hemodynamic Unit, IDIBAPS, 08036 Barcelona, Spain; nicolo.manicardi@gmail.com (N.M.); afernandezi@clinic.cat (A.F.-I.); labad@clinic.cat (L.A.-J.); maortega@clinic.cat (M.O.-R.); dsanfeliu@clinic.cat (D.S.-R.); ana3126@gmail.com (A.M.-A.); JCGARCIA@clinic.cat (J.C.G.-P.); jaume.bosch@idibaps.org (J.B.); 2 Biomedical Research Networking Center in Hepatic and Digestive Diseases (CIBEREHD), 28029 Madrid, Spain; froyo.ciberehd@cicbiogune.es (F.R.); ajhesshe@clinic.cat (A.J.H.); cfonde@clinic.cat (C.F.); juanjo.lozano@ciberehd.org (J.J.L.); fcubero@ucm.es (F.J.C.); agustin.albillos@uah.es (A.A.); j.vaquero@iisgm.com (J.V.); jfalcon@cicbiogune.es (J.M.F.-P.); 3 Center for Cooperative Research in Biosciences (CIC bioGUNE), Basque Research and Technology Alliance (BRTA), Derio, 48160 Bizkaia, Spain; mazkargorta@cicbiogune.es (M.A.); felortza@cicbiogune.es (F.E.); 4 General & Digestive Surgery, Institut Clínic de Malaties Digestives i Metabòliques, Hospital Clínic, IDIBAPS, University of Barcelona, 08036 Barcelona, Spain; 5 Hepatology, Department of Biomedical Research, Inselspital & University of Bern, 3010 Bern, Switzerland; 6 Department of Immunology, Ophthalmology and ENT, Complutense University School of Medicine, 12 de Octubre Health Research Institute (imas12), 28040 Madrid, Spain; 7 HepatoGastro Lab, Servicio de Ap. Digestivo del HGU Gregorio Marañón, Instituto de Investigación Sanitaria Gregorio Marañón (IiSGM), 28007 Madrid, Spain; 8 Department of Gastroenterology and Hepatology, Hospital Universitario Ramón y Cajal, Instituto Ramón y Cajal de Investigación Sanitaria (IRYCIS), Universidad de Alcalá, 28034 Madrid, Spain; 9 IKERBASQUE, Basque Foundation for Science, Bilbao, 48015 Bizkaia, Spain

**Keywords:** LSEC, extracellular vesicles, hepatic stellate cell, RNAseq

## Abstract

**Simple Summary:**

We define the transcriptome and secretome of primary LSECs during the progression of cirrhosis, revealing specific molecular signatures, novel biomarkers and therapeutic targets for new disease-modifying treatments for patients with advanced chronic liver disease.

**Abstract:**

The poor prognosis of chronic liver disease (CLD) generates the need to investigate the evolving mechanisms of disease progression, thus disclosing therapeutic targets before development of clinical complications. Considering the central role of liver sinusoidal endothelial cells (LSECs) in pre-neoplastic advanced CLD, the present study aimed at investigating the progression of CLD from an endothelial holistic perspective. RNAseq defined the transcriptome of primary LSECs isolated from three pre-clinical models of advanced CLD, during the progression of the disease, and from fresh human cirrhotic tissue. At each stage of the disease, the effects of LSECs secretome on neighboring cells and proteomic analysis of LSECs-derived extracellular vesicles (EVs) were also determined. CLD was associated with deep common modifications in the transcriptome of LSECs in the pre-clinical models. Pathway enrichment analysis showed predominance of genes related with pro-oncogenic, cellular communication processes, and EVs biogenesis during CLD progression. Crosstalk experiments revealed endothelial EVs as potent angiocrine effectors. The proteome of LSECs EVs showed stage-specific signatures, including over-expression of tropomyosin-1. Proof-of-principle experiments treating cirrhotic HSCs with recombinant tropomyosin-1 suggested de-activating effects. Our data provide the basis for discovering novel biomarkers and therapeutic targets for new disease-modifying treatments for patients with advanced CLD.

## 1. Introduction

Cirrhosis represents the final pre-neoplastic stage of a group of chronic liver diseases (CLD) characterized by a complex ensemble of longstanding pathophysiological processes that alters liver function, architecture and hemodynamics. Cirrhosis constitutes a major public health problem, accounting for roughly 2 million deaths per year worldwide [1].

In the last decades, many efforts have been made to understand the biological processes involved in CLD. Different studies have demonstrated how liver cells phenotype alterations generate and perpetuate a cascade of mechanisms that altogether lead to the histological distortion and microcirculatory dysfunction typical of the cirrhotic liver [2,3,4], giving rise to portal hypertension (PH), the main non-neoplastic complication of the disease [5]. Nevertheless, many studies on the underlying mechanisms of CLD and PH have been made only at the end-stage of the disease, thus ignoring the chronological changes that promote progression towards cirrhosis. In this context, animal models of liver disease represent an optimal choice when it comes to study the sequential mechanisms of these alterations [6].

The liver has four main cellular components: hepatocytes (Hep), liver sinusoidal endothelial cells (LSECs), hepatic stellate cells (HSCs), and hepatic resident macrophages (HMΦs). All these cells work in synergy, producing molecules that modulate their differentiation and activity [7] and communicating through paracrine and autocrine mechanisms to maintain liver homeostasis [8]. In particular, LSECs shape the permeable wall of the sinusoids and are actively involved in the dynamic communication process with other hepatic cells, where they promote vasoactive, inflammatory and immune functions. LSECs are pivotal regulators of liver microcirculation and fibrosis, and the maintenance of their specific phenotype generates a healthy environment in which liver sinusoids and liver function are preserved [5,9]. In the context of cirrhosis, LSECs exhibit a de-differentiated phenotype producing a vasoconstrictor, pro-thrombotic, pro-fibrogenic and pro-inflammatory milieu that negatively affects neighboring cells [10].

Every cell in the organism can produce and secrete biological materials that ultimately act as inter-cellular signaling mediators. Indeed, the cellular secretome is mainly composed by free soluble factors and extracellular vesicles (EVs), the latter ones defined as bilayered nano-sized structures that are able to envelop proteins, lipids and genetic material, shuttling this biological information between cells [11]. In the field of hepatology, different studies have evidenced the role of the hepatic secretome as mechanism of cellular communication within the liver, thus contributing to the pathophysiology of relevant diseases including alcoholic hepatitis and steatohepatitis [12,13,14,15].

Considering that chronological changes that take place within the liver sinusoid during CLD progression remain mostly unknown, and that LSECs represent a fundamental cell type regulating hepatocyte function, fibrosis progression and microcirculatory status, the present study aimed at providing a comprehensive characterization of LSECs phenotype in human cirrhosis and during cirrhosis progression in experimental models, as a way for ultimately discover novel therapeutic targets and specific biomarkers of CLD.

## 2. Materials and Methods

### 2.1. Animal Models of Liver Disease

To study the biology of the LSECs during CLD progression, twenty-seven male Wistar Han rats weighing 50–75 g underwent inhalation exposure to carbon tetrachloride (CCl_4_) for 2 weeks, 6 weeks or 12–14 weeks (*n* = 9 per group).These 3 time-points correspond respectively to acute liver injury, moderate-fibrosis and advanced cirrhosis [16].

To have a more complete approach to the heterogeneity of the CLD we included two additional models of advanced CLD. Sprague-Dawley male rats were treated with thioacetamide (TAA), administered intraperitoneally twice per week for 12 weeks as described previously [17]. Control rats received saline solution (*n* = 5 per group). In addition, secondary biliary cirrhosis was induced in Sprague-Dawley male rats by ligation of the common bile duct (cBDL) for 4 weeks as described previously [18]. In sham animals, only surgery was performed without ligation of the common bile duct (*n* = 5 per group).

Animals were kept at the University of Barcelona Faculty of Medicine facilities with controlled temperature, humidity and light/dark cycle. Animals were fed ab libitum with water, supplemented with phenobarbital (0.3 g/L) in the CCl_4_ model, and standard rodent food pellets.

In the CCl_4_ and TAA models, and to diminish acute inflammatory gene response, LSECs were isolated after 5–7 days of the last toxicant administration. All procedures were approved by the Laboratory Animal Care and Use Committee of the University of Barcelona and were conducted in accordance with the European Community guidelines for the protection of animals used for experimental and other scientific purposes (EEC Directive 86/609).

### 2.2. Rat LSECs Isolation and Secretome Fractionation

LSECs were isolated from rats at the three stages of CCl_4_-CLD progression, from TAA-cirrhotic rats, from cBDL-cirrhotic rats, and from healthy rats as previously described [19]. For detailed procedures see supplementary information.

After 16 h of culture, LSECs supernatant was collected, centrifuged at 2000× *g* for 10 min to remove cell debris and apoptotic bodies, pooled (1 pool = 3 rats) and stored at −80 °C until secretome fractionation. Secretome fractionation was performed by sequential ultra-centrifugations at increasing g force accelerations as described in previous protocols [20]. Briefly, after two ultra-centrifugation cycles performed with Sorvall WX Ultraseries Centrifuge (ThermoScientific, Waltham, MA, USA) and a P40 ST swinging bucket (Hitachi, Tokyo, Japan) four fractions were obtained: the non-ultracentrifuged supernatant (complete secretome, CS), the 10,000 g pellet enriched in large extracellular vesicles (large EVs, 150–1000 nm diameter), the 100,000 g pellet containing small extracellular vesicles (small EVs, 50–150 nm diameter) and the final extracellular vesicles-depleted supernatant containing un-pelleted soluble factors (SF). EVs enriched sub-fractions underwent routine quality control analysis to determine concentration and quantity following the MISEV 2018 guidelines [21] (see Supplementary Information).

### 2.3. Human LSECs Isolation

Human liver tissue remnants from liver resections and explants from liver transplantation surgery were used for LSECs isolation and RNAseq. Non-lesioned non-tumorous liver tissues from hepatic resections for solitary metastatic colorectal cancer were used as healthy controls and compared to cirrhotic liver explants from ethanol aetiology (*n* = 4–5 per group). Additional donor information is provided in Table S16. All patients received and signed the informed consent according to the Ethics Committee of the Hospital Clínic de Barcelona (HCB/2015/0624). hLSECs isolation procedure is detailed in supplementary information.

### 2.4. LSECs RNA-Sequencing

Human and rat LSECs RNA concentration and quality were assessed with Qubit RNA HS Assay kit (Thermo Scientific, Eugene, OR, USA) and Agilent RNA 6000 Nano chips (Agilent Technologies, Waldbronn, Germany), respectively. Samples with RIN > 8 were used for RNA sequencing. RNA libraries were prepared and amplified using Universal Plus mRNA-Seq (hLSECs; NuGEN, Leek, Netherlands) or TruSeq Stranded mRNA (rLSECs; Illumina Inc., San Diego, CA, USA) sample preparation with the corresponding kit. Libraries size around 300 bp determined using Agilent High Sensitivity DNA Chip (Agilent Technologies) were sequenced in an Illumina platform HiSeq2500. Transcriptomic analyses were performed as previously described [22]. Briefly, STAR program against *Homo sapiens* genome hg38 (gencode.v26) or *Rattus norvergicus* genome (Rnor_6.0) was used for mapping the reads followed by the quantification of genes and transcripts with the RSEM program. We used TMM method and limma-voom transformation to normalize the non-biological variability. Differential expression between different groups was assessed using moderated t-statistics [23]. Molecular processes implicated in significant deregulated genes were analyzed with Ingenuity Pathway Analysis (IPA) software (Qiagen, Hilden, Germany), and enrichment analyses of deregulated genes were performed using DAVID Bioinformatics Resources 6.8. All significant deregulated genes were considered when fold-change > 1.5 and *p*-value < 0.05. RNA sequencing data is available in GEO database: GSE164799 for human and GSE164878 for rat. Data are available at www.shiny.lvbrg.barcelona/lsec (accessed on 14 May 2021).

### 2.5. Rat Hepatocytes, HSCs, HMΦ In Vitro Crosstalk

Hepatocytes (5 × 10^5^ cells/mL), HSCs (80% confluence) and HMΦs (80% confluence) isolated from healthy rats were incubated with LSECs’ secretome fractions from 3 different biological pools (per each CLD stage) at the following concentrations: CS 550 ng/μL, large EVs 30 ng/μL, small EVs 30 ng/μL and SF 400 ng/μL. Concentrations were established considering bibliography and the biological fluctuation of the protein amount of EVs during the progression of CLD in the rat model. CS and SF fractions were adjusted to the minimum quantity of protein detected in all pools (110 μg and 80 μg respectively) giving the final concentrations above mentioned. After 24 h incubation, cells were collected with RLT buffer containing 10 µL/mL of β-mercaptoethanol (63689–25 ML-F, Sigma, St. Louis, MO, USA) for subsequent RNA analysis.

### 2.6. LSECs Secretome Proteomics

Proteomic analysis of LSECs’ secretome was performed at the Proteomic platform of CIC bioGUNE (Derio, Spain) using a novel hybrid trapped ion mobility spectrometry— quadrupole time of flight mass spectrometer (tims TOF Pro with PASEF, Bruker Daltonics; Billerica, MA, USA) coupled online to a nanoElute liquid chromatograph (Bruker) as detailed in the Supplementary Information.

### 2.7. Small EVs Internalization Assay

Small EVs from cirrhotic rat LSECs (15 ng/µL) were labelled with the fluorescent lipophilic dye PKH26 (MINI26–1 KT, Sigma-Aldrich, St Quentin Fallavier, Lyon) according to manufacturer’s protocol, with minor modifications, and added to the supernatant of healthy rat HSCs during 1 and 4 h. After the respective incubation periods, HSCs were washed thrice with PBS and fixed with 2% PFA in PBS and mounted with Fluoromont-G (E2220-RB70, Southern Biotech, Birmingham, AL, USA) onto microscope slides (J1800 AMNZ, Thermo Scientific). Internalized vesicles were captured with a BX41 microscope (Olympus, Tokyo, Japan) coupled to a U-RFL-T burner (Olympus). Color channels were merged and balanced using ImageJ software (Fiji, Madison WI, USA). Cells nuclei were stained with the NucBlue™ reagent (R37605, Invitrogen, Waltham, MA, USA). Images were taken at 400× magnification.

### 2.8. Recombinant Tropomyosin-1 In Vitro Assay

Homology between rat and human TPM1 aminoacidic sequences was determined using the Protein Blast server (NCBI, Bethesda, MD, USA) resulting in a 94% homology between sequences. Human recombinant TPM1 (LS-G905, LSBio, Seattle, WA, USA) was used to assess its activity on primary isolated rat HSCs. HSCs were treated with three different concentrations of TPM1 (0.1, 1, and 10 µg/mL). After 24 h cells were lysed for RNA extraction and real time RT-PCR was performed as described in supplementary information.

### 2.9. Protein Expression Analyses

Protein (immunofluorescence and Western blot) assays are further described in supplementary information.

### 2.10. Statistical Analysis

Statistical analysis was performed with Prism 8 (GraphPad Software, La Jolla, CA, USA). All results are expressed as mean ± S.E.M. Comparisons between groups were performed with t test or Mann-Whitney U test for non-parametric variables. Differences were considered significant at a *p* value < 0.05. Proteome statistics were performed as detailed in supplementary information.

## 3. Results

### 3.1. Rat Cirrhotic LSECs Transcriptome

**Figure 1:**
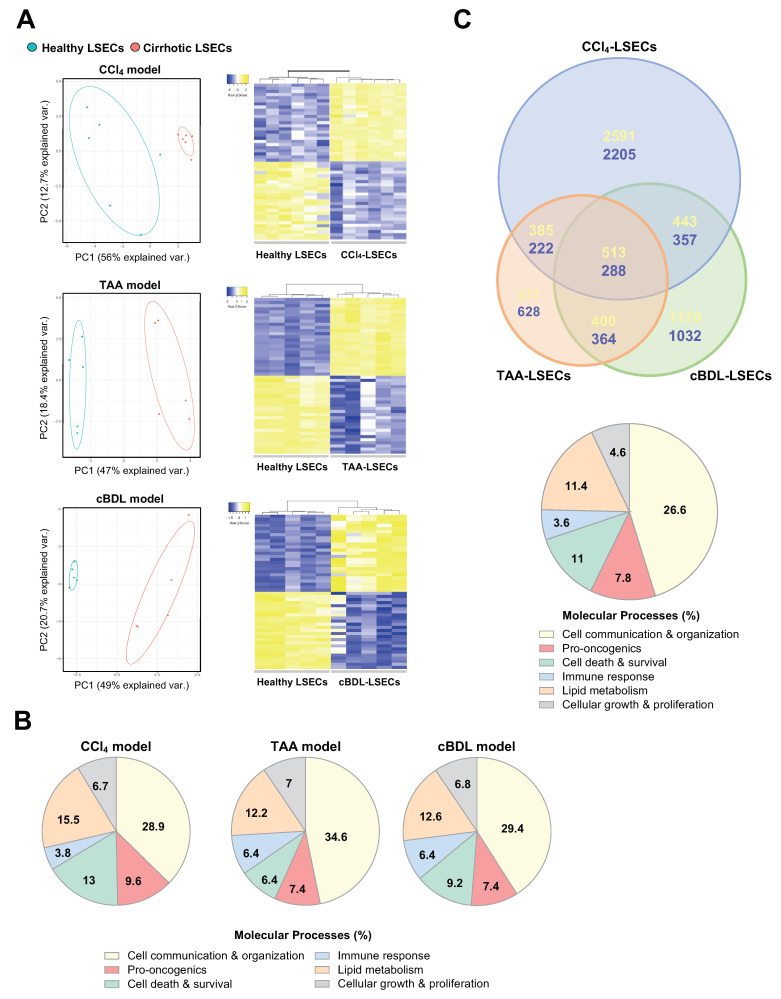
LSECs transcriptome in experimental models of advanced CLD. (**A**) Transcriptomic profile of LSECs isolated from CCl_4_, TAA and cBDL pre-clinical models of chronic liver disease and healthy animals represented as Principal Component Analysis graphic (Left), and top 25 up- (yellow) and down-regulated (blue) genes represented in heatmaps (Right). (**B**) Molecular processes deregulated in cirrhotic LSECs analysed by Ingenuity Pathway Analysis software. (**C**) Venn diagram showing the commonly up-regulated (yellow) or down-regulated (blue) genes comparing three pre-clinical models of CLD (Up), and main molecular processes that define the common differentially expressed genes (Bottom). *n* = 5–6 independent LSECs isolations per experimental model & healthy. Significant differences are considered in RNAseq when fold-change > 1.5 and *p*-value < 0.05.

RNA sequencing of LSECs isolated from cirrhotic CCl_4_ (12–14 weeks), TAA and cBDL pre-clinical models of cirrhosis identified 7028 differentially expressed genes (DEG) in cirrhotic CCl_4_-LSECs (56.2% up-regulated and 43.8% down-regulated) (Table S1), 3382 DEG in cirrhotic TAA-LSECs (55.6% up-regulated and 44.4% down-regulated) (Table S2), and 4578 DEG in cBDL-LSECs (55.4% up-regulated and 44.6% down-regulated) (Table S3) compared with their corresponding healthy LSECs (fold-change > 1.5 and *p*-value < 0.05) (Figure 1A).

DEG in each model were then analyzed using the IPA software. The main molecular processes defined in cirrhotic LSECs de-regulation were cell communication and organization, pro-oncogenic processes, cell death and survival, immune response, lipid metabolism, and cellular growth and proliferation, among others (Figure 1B). These results suggested that cell signaling and communication is key in LSECs pathobiology in cirrhosis development.

Although the number of total DEG was higher in CCl_4_-LSECs, a large number of de-regulated genes (801) were commonly shared in the three pre-clinical models (Figure 1C, Figure S1A,B and Table S4). Additional analysis of the shared DEG in LSECs from experimental models using the DAVID Bioinformatics database [24] distinguished that 140 genes (17.6%) were involved in cellular components of EVs and their biogenesis (Figure S1C and Table S5).

### 3.2. Human Cirrhotic LSECs Transcriptome

**Figure 2:**
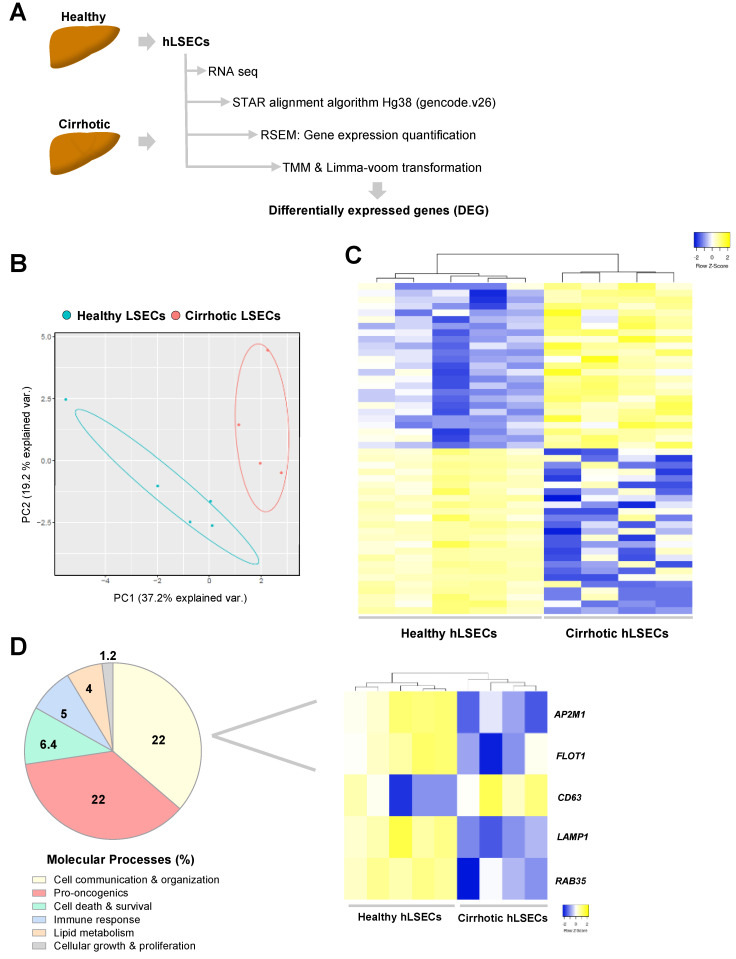
Transcriptomic profile of human LSECs in advanced CLD. (**A**) LSECs were isolated from human healthy and cirrhotic liver tissue, RNA was sequenced, and differential expression of genes was obtained as illustrated. (**B**) Principal Component Analysis of healthy and cirrhotic human LSECs transcriptomic profile. (**C**) Top 25 of up- (yellow) and down-regulated (blue) human cirrhotic LSECs genes depicted in heatmap. (**D**) Left, molecular processes deregulated in human cirrhotic LSECs analysed by Ingenuity Pathway Analysis. Right, heatmap of representative genes involved in extracellular vesicles biogenesis and secretion. *n* = 5 healthy LSECs and *n* = 4 cirrhotic LSECs. Significant differences are considered in RNAseq when fold-change > 1.5 and *p*-value < 0.05.

The transcriptome of healthy and cirrhotic human CD32 b+ LSECs was analyzed using bulk RNA sequencing as resumed in Figure 2A. Similarly to the data from pre-clinical models, the principal component analysis (PCA) defined two clusters of LSECs according to healthy or cirrhotic phenotype (Figure 2B). After TMM and limma transformation, cirrhotic-LSECs exhibited 1411 DEG with fold-change > 1.5 and *p*-value < 0.05 compared with healthy LSECs (48.8% up-regulated and 51.2% down-regulated). As shown in Figure 2C, DEG were grouped in two main patterns, healthy or cirrhotic LSECs, demonstrating that cirrhotic LSECs showed different transcriptomic profile compared to healthy. Top 25 up- and down-regulated genes are described in Table S6.

Molecular processes analysis of DEG in human cells using IPA software confirmed “cell communication & organization” as a key process implicated in LSECs capillarization in cirrhosis (Figure 2D, Left). Indeed, various genes implicated in formation and excretion of EVs were significantly modulated in cirrhotic hLSECs as shown in the heatmap (Figure 2D, Right).

Gene ontology analysis between common DEG in human cirrhotic and LSECs isolated from different pre-clinical models of CLD revealed that a remarkable % of genes defined extracellular EVs as one of the main cellular components (Supplementary Figure S2). Specifically, human cirrhotic cells shared 71 genes (27.6%) with CCl_4_-LSECs (Tables S7 and S8), 43 genes (32%) with TAA-LSECs (Tables S9 and S10), 79 genes (28.6%) with cBDL-LSECs (Tables S11 and S12) and eight genes (24.3%) with the three experimental models of CLD implicated in EVs components. These transcriptomic analyses further suggested that cell communication through EVs represents an important process both in human cirrhotic and animal cirrhotic LSECs capillarization.

### 3.3. LSECs Transcriptomic Profile during Cirrhosis Progression

**Figure 3:**
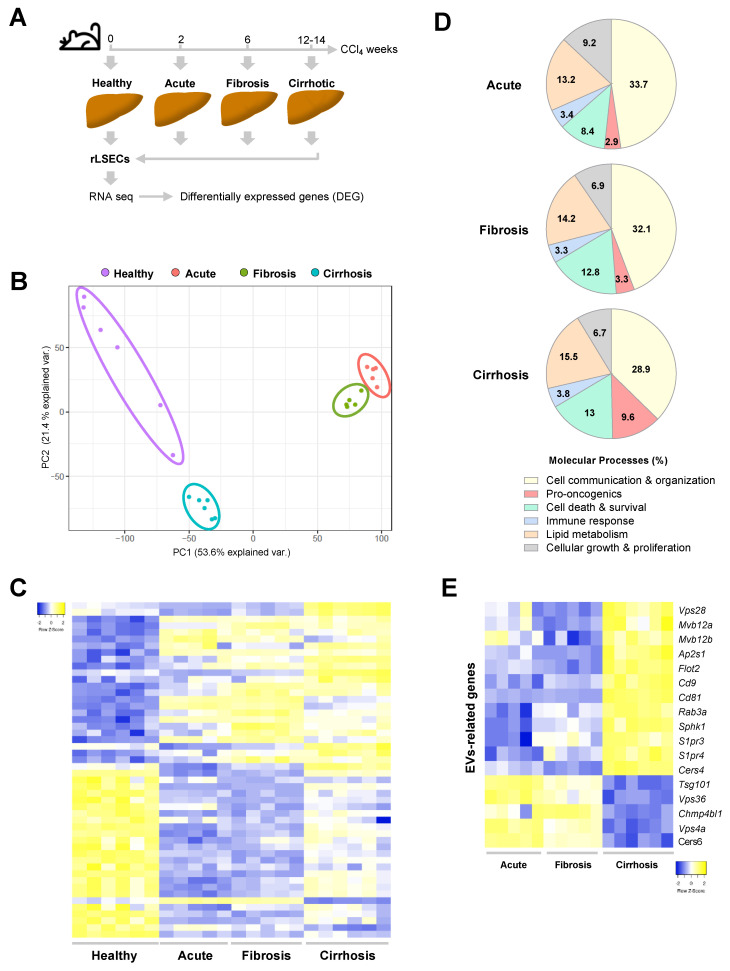
LSECs transcriptome during the progression of CLD. (**A**) Schematic diagram of LSECs isolation and RNAseq during CLD progression. (**B**) Principal Component Analysis representation of LSECs isolated at different stages of liver disease. (**C**) Top 25 up- (yellow) and down-regulated (blue) LSECs genes during CLD progression. Deregulated genes have been ordered according to the fold-change in cirrhotic LSECs. (**D**) Molecular processes of LSECs deregulated genes in each stage of CLD analysed by Ingenuity Pathway Analysis. (**E**) Representative deregulated genes during CLD progression implicated in the biogenesis and exocytosis of extracellular microvesicles. *n* = 5–6 independent LSECs isolations per disease stage. Differences in RNAseq are considered significant when fold-change > 1.5 and *p*-value < 0.05.

Progressive CCl_4_ administration periods defined three phases of liver injury in the rat model namely acute (after 2 weeks), fibrotic (6 weeks of administration) and decompensated cirrhosis when rats developed ascites (12–14 weeks) (Figure 3A). LSECs were isolated from healthy and CCl_4_-treated rats, the RNA was sequenced, and the transcriptome profile was analyzed. As shown in Figure 3B, LSECs populations were clustered in four groups after performing PCA. Transcriptomic analyses showed 9148 DEG (47.5% up-regulated and 52.5% down-regulated) in LSECs after acute liver injury and 8813 at the stage of liver fibrosis (48.5% up-regulated and 51.5% down-regulated) compared to healthy-LSECs with a fold-change > 1.5 and *p*-value < 0.05 (Figure 3C and Supplementary Table S13). The results of cirrhotic LSECs are explained above (Section 3.1). We next characterized the transcriptomic results using IPA in each stage. LSECs showed differential transcriptomic profile during cirrhosis progression; however, processes implicated in cell communication and cellular organization are remarkable during all the disease progression, from acute liver injury until advanced cirrhosis, representing around of 30% of the total molecular processes involved in genes de-regulation (Figure 3D). Concretely, we found that several genes related to molecular complexes and membrane receptors necessary for EVs biogenesis and exocytosis were differentially expressed in LSECs from the three stages of CCl_4_ rat model when compared to healthy LSECs (Figure 3E and Supplementary Table S14). Notably, genes related with pro-oncogenic processes were incrementally de-regulated during CLD progression (Figure 3D).

### 3.4. Effects of LSECs Secretome on Hepatic Neighboring Cells during CLD

Complete secretome (CS), or conditioned media, was obtained from primary LSECs isolated from rats at the three stages of CLD. To avoid a potential source of exogenous EVs contamination, we opted for an EVs-depleted FBS and endothelial cell growth supplement was not added to cell cultures, guaranteeing therefore a pure rat LSECs-derived secretome [21]. Since our interest was to observe the effect of the complete secretome on neighboring liver cells, LSECs medium was also not complemented with heparin which could mask EVs receptors on recipient cells [25].

Recipient cells (Hep, HSCs and HMΦs) from healthy rats were incubated with LSECs CS from acute, fibrotic and cirrhotic stages and the expression of a specific gene set for each recipient cell type was assessed. Hnf4α, Alb, Slc22 a1 and Slc10 a1 gene expression were assessed as markers of hepatocyte phenotype [26], Col1α1 and Pdgfrβ as markers of HSCs activation and collagen producing phenotype [27], Mrc1 and Il-10 for HMΦ pro-resolutive phenotype and Il-6 and Il-1β for HMΦ pro-inflammatory phenotype [28].

**Figure 4:**
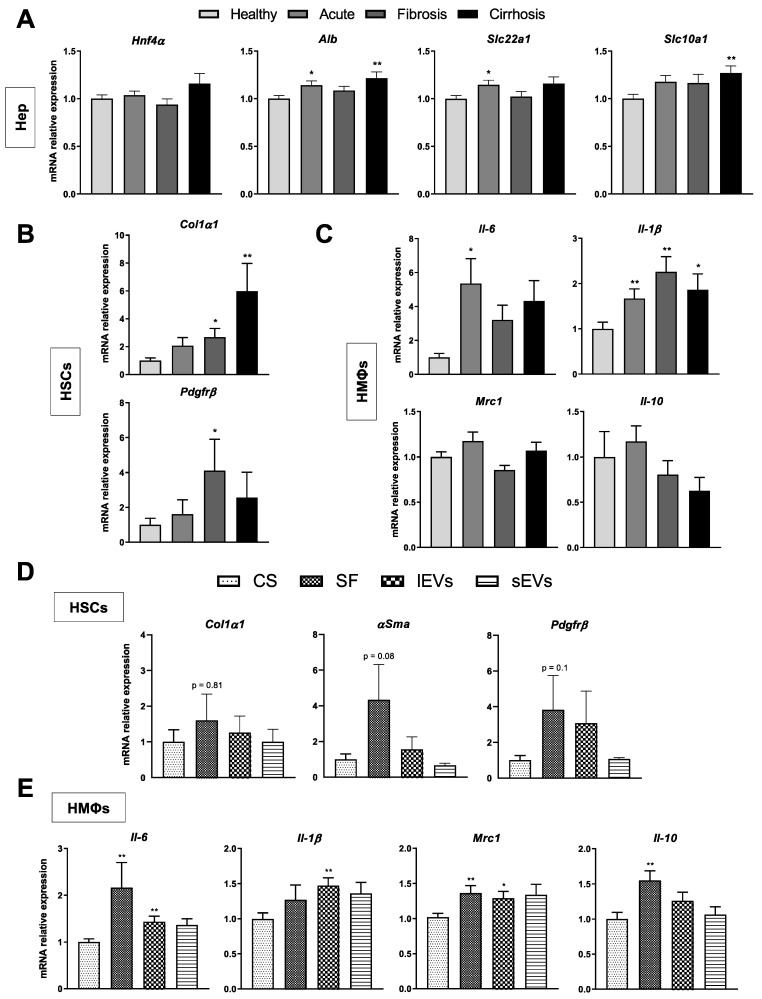
Effects of LSECs secretome on neighbouring liver cells during CLD. mRNA expression of specific phenotype markers in hepatocytes (**A**), HSCs (**B**) and HMΦs (**C**) incubated for 24 h with the complete secretome (CS) of LSECs isolated at each stage of CLD. mRNA expression of phenotype markers in HSCs (**D**) and HMΦs (**E**) after 24 h of incubation with the different sub-fractions of cirrhotic LSECs secretome: large extracellular vesicles (lEVs), small EVs (sEVs) and soluble factors (SF), normalized to CS-receiving cells. Results derive from *n* = 3–5 independent experiments. * *p* < 0.05 and ** *p* < 0.01 on t test (Mann-Whitney U test for non-parametric variables) for differences between each group and the CS group.

LSECs CSs from specific liver disease stages affected neighboring cells in different ways. Hepatocytes were minimally affected by LSEC secretomes (Figure 4A) while HSCs and HMΦs were mostly modulated by CSs proceeding from fibrotic and cirrhotic LSECs. Indeed, CSs from fibrotic and cirrhotic stages induced an HSC proliferative and collagen producing phenotype, with no significant changes in αSMA, while those from an acute stage of CLD were not able to induce the same phenotype switch (Figure 4B). Pro-activation effects of cirrhotic.

LSEC CS were further confirmed by augmented expression of αSMA protein in HSCs when incubated for 48 h (Supplementary Figure S3). CS from cirrhotic stage also affected HMΦs that polarized toward a pro-inflammatory phenotype with no changes in those genes related to the HMΦs pro-resolutive phenotype (Figure 4C).

### 3.5. Effects of LSECs Secretome Sub-Fractions on Liver Cells Phenotype

Once established the disease progression time point at which phenotypic changes occur in neighboring cells, we wanted to understand whether the observed changes were due to the entire secretome or to a precise sub-fraction of it. We assessed the activity of secretome fractions principally on HSCs and HMΦs, since hepatocytes appeared barely affected by CS.

Although EVs physiological concentration is still unknown, we used the concentration of EVs normally used in in vitro experiments [29,30]. We also analyzed the concentration of the large EVs and small EVs fractions, the presence of the principal EVs-related markers by immunoblot, and finally visualized small EVs by Cryo-TEM (Supplementary Figure S4).

Regarding HSCs, we did not observe a predominant secretome sub-fraction promoting stellate cell activation (Figure 4D), but HMΦs gene expression showed a slightly greater effect of the SF and large EVs fractions on both the group of genes related to the pro-inflammatory and pro-resolutive phenotype, suggesting that molecular components shuttled in these fractions may be prone to rapidly prepare the HMΦ cells machinery to both inflammatory and resolutive cellular strategies (Figure 4E).

### 3.6. LSECs Secretome Profile during Cirrhosis Progression

Next, we set out to characterize the components of the LSECs secretome during CLD. We decided to thoroughly investigate the sub-fraction corresponding to small EVs for various reasons: first, because of their relevance and growing attention in the field of Hepatology [11], where LSECs EVs cargo has never been characterized; second, because of their potential importance in future therapeutic developments and use as disease biomarkers [31]; and finally, because we observed a change in the pattern of released EVs by cirrhotic LSECs, with a decrease in large EVs associated with a concomitant increase in small EVs levels (Supplementary Figure S4A).

We first confirmed the ability of HSCs to internalize small EVS from cirrhotic LSECs (Supplementary Figure S5) and subsequently performed a proteomic characterization of the small EVs LSECs-borne fraction across CLD. The proteomic analysis detected 1034 proteins of which 345 were submitted to further analysis. Such reduction reflects the selection criteria used to ensure that the proteins detected were directly coming from cultured rat LSECs and not from any contamination source (see supplementary information).

**Figure 5:**
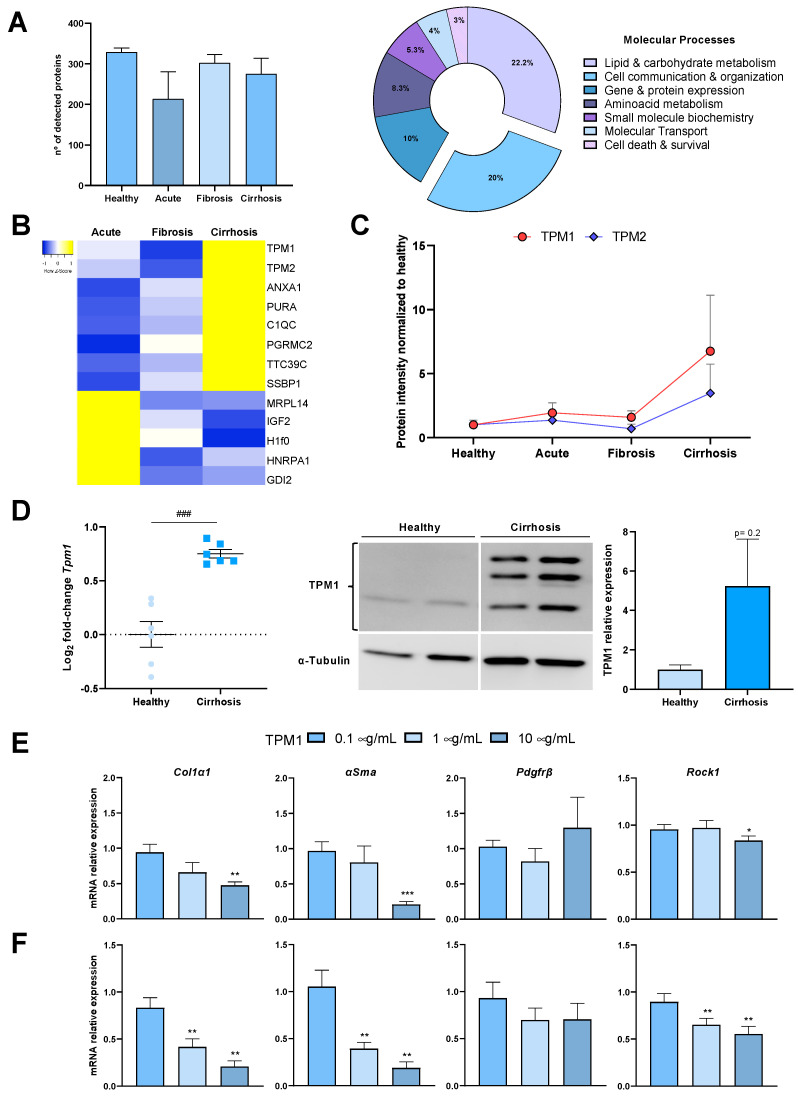
LSECs extracellular vesicles proteomics during CLD progression. (**A**) Left, number of proteins detected in LSECs-derived small EVs at each stage of CLD progression. Right, cellular processes of deregulated proteins in cirrhotic LSECs EVs analysed by Ingenuity Pathway Analysis. (**B**) Heatmap of the top up- (yellow) and down-regulated (blue) proteins of LSECs EVs during CLD progression and, (**C**) graphical representation of Tpm1 & 2 gene expression in LSECs EVs across CLD. (**D**) Left, mRNA expression of Tpm1 in healthy and CCl_4_-cirrhotic LSECs. Values are represented in log2 fold-change. Right, western blot representative image of TPM1 in healthy and CCl_4_-cirrhotic LSECs, and corresponding quantification. (**E**) mRNA expression of depicted genes in in vitro activated HSCs and (**F**) primary isolated cirrhotic CCl4-HSCs exposed to different concentrations of recombinant TPM1 for 24 h. Red dot lines represent vehicle-treated cells. Results derive from *n* = 9 isolations (**A**,**B**), *n* = 9 independent samples (**C**), *n* = 6 independent isolations (**D**) and *n* = 3–5 independent experiments (**E**,**F**). ### *p* < 0.001 t test between cirrhotic and healthy group; * *p* < 0.05, ** *p* < 0.01 and *** *p* < 0.001, t test (Mann-Whitney U test for non-parametric variables) for differences between each group and the corresponding control group.

Of the 345 proteins, 329 were detected in the healthy group, 213 in the acute injury group, 303 in the fibrotic group and 275 in the cirrhotic group (Figure 5A, Left). We evaluated the relative expression of the detected proteins using the Perseus platform [32] and ran an IPA to assess which biological processes the detected proteins were mostly related to. Most of the IPA matches were with the categories of cellular organization and communication, lipid and carbohydrate metabolism and gene and protein expression, among others (Figure 5A, Right). 

Within the analyzed proteins we found various molecules of potential interest such as histones known to act as DAMPs [33], and elements of the cellular cytoskeleton and cellular contractile machinery such as tropomyosins, specifically TPM1 and TPM2 (Figure 5B, Supplementary Table S15). In the case of TPM1, the IPA software grouped it within the categories of cellular assembly, organization and signaling and the relative expression analysis with Perseus detected a specific augmented expression of the protein in the cirrhotic stage while maintaining a moderate expression in the acute and fibrotic stages when compared with the healthy group (Figure 5C). Consistent with this observation, mRNA and protein expression of TPM1 was up-regulated in CCl_4_-cirrhotic LSECs (Figure 5D).

### 3.7. Effects of TPM1 on HSCs Phenotype

Considering the differential expression of TPM1 in cirrhotic LSECs small EVs, and the angiocrine potential of this protein [34], we examined the effects of TPM1 on primary rat activated HSCs. First, we used in vitro activated HSCs observing a significant decrease in the gene expression of Col1α1, αSma and Rock1 when treating them with 10 µg/mL, with no major differences in Pdgfrβ expression when compared to vehicle (Figure 5E). The same findings were observed in HSCs freshly isolated from cirrhotic rats (Figure 5F). No signs of HSCs death were observed in response to treatments.

## 4. Discussion

In the present study, we approached LSECs pathophysiological impact on CLD by analyzing its phenotypic modifications during the progression to cirrhosis of the liver, the pre-neoplastic stage of liver cancer [35]. LSECs are known to elicit a pivotal role in the maintenance and regulation of the liver milieu. Indeed, they possess a unique phenotype and are involved in the regulation of multiple biological processes such as angiogenesis, vascular regulation of sinusoidal blood flow, monitoring of gut microbial products and regulation of the immune response along with HMΦs [36,37]. They constitute the liver’s first line of defense and the injury-borne endothelial dysfunction has been described as a main driver of the processes related to HSCs activation and the establishment of PH [5]. Because of this, understanding the grades of LSECs dysfunctionality during the progressive development of CLD may help at defining novel pathobiological pathways and therapeutic opportunities for cirrhosis.

To pursue this aim we first characterized the transcriptome of LSECs isolated from three widely used pre-clinical models of advanced CLD, then, validated these pre-clinical results with data from primary human cirrhotic LSECs and, finally, developed a progressive liver injury model to mimic the chrono-biological alterations of CLD we wanted to investigate. Transcriptomic analysis revealed valuable data regarding the genes and the molecular pathways de-regulated in advanced CLD, disclosing those related with oncogenic processes and cellular communication as the two major descriptors of the rat capillarized liver endothelium. The same molecular processes defined the transcriptome of human CD32 b+ LSECs isolated from patients with CLD compared to controls. Detailed analysis of de-regulated genes in our transcriptomic data revealed that most of the deregulated genes both in human and rat cirrhotic LSECs were related to communication processes, specifically with the biogenesis and exocytosis of EVs. This observation agrees with previous studies demonstrating that LSECs paracrinally communicate with other resident liver cells being able, for example, to regulate HSCs phenotype [9,38,39]. Moreover, our RNAseq data coincide with recent works characterizing the liver endothelium at single cell level [38,39,40]. Indeed, molecular processes and cellular component analyses of previous human and mouse transcriptomic data confirmed that vesicle-mediated transport and cell communication and organization molecular processes are markedly induced in cirrhotic LSECs (Supplementary Figure S6), overall validating our observations and reinforcing the importance of LSECs-mediated cross-talk within the liver milieu.

We then wondered whether LSECs-derived EVs could convey specific biological information that changes along the progression of CLD and that may have a stage-dependent influence on the phenotype of neighboring liver cells. To test this hypothesis, we used a pre-clinical model of CLD, where stage-specific changes in LSECs phenotype and secretome can be investigated. Particularly, the transcriptome and secretome of the liver endothelium was characterized across the progression of CLD using the CCl_4_ pre-clinical model of cirrhosis. This animal model has been used to characterize stage-specific changes in CLD disease [16] and probably represents the mostly used experimental model to investigate the pathophysiology of CLD and its clinical complications. Molecular pathways analysis of deregulated genes in each stage of the disease revealed that approximately one third of them relate to cellular interaction/communication, confirming the importance of LSEC angiocrine activity during chronic liver injury. On the other hand, fluctuation of certain pathways along cirrhosis progression was observed, including a reduction in immune response and an increment in pro-oncogenic genes, which indeed deserves future investigations that are out of the scope of the present work.

Analysis of the genes involved in LSEC angiocrine capacity, specifically in the biogenesis of EVs, showed a stage-dependent up-regulation in the ESCRT-dependent pathway (Mvb12 a, Mvb12 b), lipid-rafts structures such as Flot2, the tetraspanins Cd9 and Cd81, and the Rab3 a small GTPase responsible for migration of EVs to the plasma membrane and subsequent exocytosis, among others. Although such up-regulations were not accompanied by an enhanced vesicle secretion by LSECs, the crosstalk experiments on HSCs and HMΦs with the endothelial secretome fractions, suggested that the content rather than the quantity might be involved in the phenotype regulation of recipient cells. Indeed, it has been shown that EVs cargo is representative of the cell physiological stage and their content is able to sensitize the recipient cells reprogramming their behavior within the liver milieu [41].

We therefore characterized the protein content of the endothelial small EVs sub-fraction during the progression of CLD. This proteomic analysis revealed a group of potentially interesting proteins but, due to their implication in contractility processes and as a proof-of-concept study, we focused on tropomyosins and specifically on TPM1 which expression across the CLD was lightly up-regulated and finally spiked in the cirrhotic stage. TPM1 expression has been previously found in activated HSC colocalizing with αSMA in liver tissue samples from rat livers [42], and has also been detected in serum and liver tissue from patients [43], where it has been proposed as potential biomarker of cirrhosis. Despite the abovementioned studies, our results suggest that TPM1 may have a protective-like effect on activated HSCs.

We admit limitations in our study such as the use of immunomagnetism to isolate human LSECs, which may induce selection of certain cellular sub-populations. Although CD32 b has been previously used as marker for LSECs isolation [44,45], we applied gene set enrichment analysis to corroborate the phenotypic concordances between our cells and the endothelial cells described in [39] using single cell analysis. The results of these analyses confirmed that CD32 b+ cells used in our study were mostly enriched in LSECs. The number of human donors included in the study may be considered low, nevertheless it is larger than previous works and, importantly, both the molecular processes and the gene ontology analyses of human and rat cirrhotic LSECs validated the transcriptomic profile involved in human LSECs deregulation. We also admit that the results from the proof-of-concept experiments using TPM1 may be controversial. The real impact of endothelial-derived TPM1 embedded in small EVs on neighboring cells in a multi-cellular milieu, and its underlying mechanisms, remains conjectural. Future desirable studies will help to clarify the specific role of LSECs-derived EVs in CLD pathophysiology, and their possible role as novel biomarkers of endothelial dysfunction in disease.

On the other hand, we do believe that our work adds significant pieces of relevant data to the liver vascular pathobiology field. The analysis of LSECs transcriptome in three mostly used pre-clinical models of CLD provides data pointing to the singularity of each model, the commonly shared de-regulated pathways and, importantly, their similarity to the human liver endothelium in cirrhosis. It is important to highlight that deep analysis of our data demonstrated that certain genes and molecular pathways de-regulated in cirrhotic human LSEC are mirrored or not in a particular animal model, thus providing essential information for a better use of pre-clinical models of disease in order to improve the translatability of pre-clinical findings to the real clinical scenario [6]. Our data also complement previous transcriptomic works characterizing the transcriptome of sinusoidal cells in healthy [40,41,46] and CLD [40,47] scenarios, nevertheless this is the first time that the dynamic changes of LSECs transcriptome have been investigated across CLD, compared to human cells, and holistically explored considering LSECs secretome, altogether helping to further understand the pathobiology of liver endothelial dysfunction in cirrhosis.

## 5. Conclusions

In conclusion, the present study is the first to provide a comprehensive transcriptomic analysis of LSECs, and to characterize the secretory machinery of this cell type, during the progression of CLD. Although experimental in nature, we believe that our data will serve as conceptual basis for the discovery of novel biomarkers and therapeutic targets for the treatment of patients with cirrhosis and other liver diseases coursing with endothelial dysfunction.

## Data Availability

Data is contained within the article or supplementary material. RNA sequencing data are available in corresponding GEO database, and at www.shiny.lvbrg.barcelona/lsec (accessed on 14 May 2021).

## Supplementary Materials

The following are available online at https://www.mdpi.com/article/10.3390/cancers13112688/s1, Supplementary Materials and Methods. Figure S1: Common deregulated genes in CCl_4_-, TAA- and cBDL-cirrhotic LSECs. Figure S2: Cellular component analysis of commonly deregulated genes. Figure S3: Effects of cirrhotic LSECs secretome in rat HSCs. Figure S4: LSECs extracellular vesicles characterization. Figure S5: Extracellular vesicles internalization by HSCs. Figure S6: Primary LSECs isolation. Figure S7: Western blot images. Table S1: Top 25 up- & down-regulated genes in CCl_4_-cirrhotic rat LSECs. Table S2: Top 25 up- & down-regulated genes in TAA-cirrhotic rat LSECs. Table S3: Top 25 up- & down-regulated genes in cBDL-cirrhotic rat LSECs. Table S4: Top common up- and down-regulated genes between CCl_4_-, TAA and cBDL cirrhotic rat LSECs. Table S5: Common DEG between CCl_4_-, TAA- and cBDL-cirrhotic rat LSECs defining extracellular exosomes components. Table S6: Top 25 up- & down-regulated genes in human cirrhotic CD32 b+ LSECs. Table S7: Top common up- and down-regulated genes between human cirrhotic CD32 b+ LSECs and CCl_4_-cirrhotic rat LSECs. Table S8: Common DEG between cirrhotic human and CCl_4_-cirrhotic rat LSECs defining extracellular exosomes components. Table S9: op common up- and down-regulated genes between human cirrhotic CD32 b+ LSECs and TAA- cirrhotic rat LSECs. Table S10: Common DEG between cirrhotic human and TAA-cirrhotic rat LSECs defining extracellular exosomes components. Table S11: Top common up- and down-regulated genes between human cirrhotic CD32 b+ LSECs and cBDL- cirrhotic rat LSECs. Table S12: Common DEG between cirrhotic human and cBDL-cirrhotic rat LSECs defining extracellular exosomes components. Table S13: Top 25 up- & down-regulated genes in CCl_4_-cirrhotic rat LSECs during CLD progression. Table S14: Genes related to molecular complexes and membrane receptors necessary for EVs biogenesis and exocytosis. Table S15: Top up- & down-regulated proteins in CCl4-cirrhotic rat LSECs sEVs during CLD progression. Table S16: Clinical characteristics. Table S17: Primary Antibodies. Table S18: RT-qPCR primers.

## References

[1] Marcellin P., Kutala B.K. (2018). Liver diseases: A major, neglected global public health problem requiring urgent actions and large-scale screening. Liver Int..

[2] Deleve L.D. (2009). The hepatic sinusoidal endothelial cell: Morphology, function, and pathobiology. The Liver.

[3] Rockey D.C. (2006). Hepatic fibrosis, stellate cells, and portal hypertension. Clin. Liver Dis..

[4] Bernsmeier C., van der Merwe S., Périanin A. (2020). Innate immune cells in cirrhosis. J. Hepatol..

[5] Gracia-Sancho J., Marrone G., Fernández-Iglesias A. (2019). Hepatic microcirculation and mechanisms of portal hypertension. Nat. Rev. Gastroenterol. Hepatol..

[6] Nevzorova Y.A., Boyer-Diaz Z., Cubero F.J., Gracia-Sancho J. (2020). Animal models for liver disease – A practical approach for translational research. J. Hepatol..

[7] Marrone G., Shah V.H., Gracia-Sancho J. (2016). Sinusoidal communication in liver fibrosis and regeneration. J. Hepatol..

[8] Greuter T., Shah V.H. (2016). Hepatic sinusoids in liver injury, inflammation, and fibrosis: New pathophysiological insights. J. Gastroenterol..

[9] Deleve L.D., Wang X., Guo Y. (2008). Sinusoidal endothelial cells prevent rat stellate cell activation and promote reversion to quiescence. Hepatology.

[10] Xie G., Wang X., Wang L., Wang L., Atkinson R.D., Kanel G.C., Gaarde W.A., DeLeve L.D. (2012). Role of differentiation of liver sinusoidal endothelial cells in progression and regression of hepatic fibrosis in rats. Gastroenterology.

[11] Hirsova P., Ibrahim S.H., Verma V., Morton L.A., Shah V.H., LaRusso N.F., Gores G.J., Malhi H. (2016). Extracellular vesicles in liver pathobiology: Small particles with big impact. Hepatology.

[12] Szabo G., Momen-Heravi F. (2017). Extracellular vesicles in liver disease and potential as biomarkers and therapeutic targets. Nat. Rev. Gastroenterol. Hepatol..

[13] Eguchi A., Lazaro R.G., Wang J., Kim J., Povero D., Willliams B., Jiaohong W., Stärkel P., Schnabl B., Ohno-Machado L. (2017). Extracellular vesicles released by hepatocytes from gastric infusion model of alcoholic liver disease contain a MicroRNA barcode that can be detected in blood. Hepatology.

[14] Povero D., Yamashita H., Ren W., Subramanian M.G., Myers R.P., Eguchi A., Simonetto D.A., Goodman Z.D., Harrison S.A., Sanyal A.J. (2020). Characterization and proteome of circulating extracellular vesicles as potential biomarkers for NASH. Hepatol. Commun..

[15] Payancé A., Silva-Junior G., Bissonnette J., Tanguy M., Pasquet B., Levi C., Roux O., Nekachtali O., Baiges A., Hernández-Gea V. (2018). Hepatocyte microvesicle levels improve prediction of mortality in patients with cirrhosis. Hepatology.

[16] Gracia-Sancho J., Russo L., García-Calderó H., García-Pagán J.C., García-Cardeña G., Bosch J. (2010). Endothelial expression of transcription factor Kruppel-like factor 2 and its vasoprotective target genes in the normal and cirrhotic rat liver. Gut.

[17] Boyer-Diaz Z., Domingo J.C., De Gregorio E., Manicardi N., Aristu-Zabalza P., Cordobilla B., Abad-Jordà L., Ribera M.O., Fernández-Iglesias A., Marí M. (2019). A nutraceutical rich in docosahexaenoic acid improves portal hypertension in a preclinical model of advanced chronic liver disease. Nutrients.

[18] Boyer-Diaz Z., Aristu-Zabalza P., Andrés-Rozas M., Robert C., Ortega-Ribera M., Fernández-Iglesias A., Broqua P., Junien J.-L., Wettstein G., Bosch J. (2021). Pan-PPAR agonist lanifibranor improves portal hypertension and hepatic fibrosis in experimental advanced chronic liver disease. J. Hepatol..

[19] Fernández-Iglesias A., Ortega-Ribera M., Guixé-Muntet S., Gracia-Sancho J. (2018). 4 in 1: Antibody-free protocol for isolating the main hepatic cells from healthy and cirrhotic single rat livers. J. Cell. Mol. Med..

[20] Théry C., Amigorena S., Raposo G., Clayton A. (2006). Isolation and characterization of exosomes from cell culture supernatants and biological fluids. Curr. Protoc. Cell Biol..

[21] Théry C., Witwer K.W., Aikawa E., Alcaraz M.J., Anderson J.D., Andriantsitohaina R., Antoniou A., Arab T., Archer F., Atkin-Smith G.K. (2018). Minimal information for studies of extracellular vesicles 2018 (MISEV2018): A position statement of the International Society for Extracellular Vesicles and update of the MISEV2014 guidelines. J. Extracell. Vesicles.

[22] Pietrosi G., Fernández-Iglesias A., Pampalone M., Ortega-Ribera M., Lozano J.J., García-Calderó H., Abad-Jordà L., Conaldi P.G., Parolini O., Vizzini G. (2020). Human amniotic stem cells improve hepatic microvascular dysfunction and portal hypertension in cirrhotic rats. Liver Int..

[23] Smyth G.K. (2005). limma: Linear Models for Microarray Data. Bioinformatics and Computational Biology Solutions Using R and Bioconductor.

[24] Huang da W., Sherman B.T., Lempicki R.A. (2009). Systematic and integrative analysis of large gene lists using DAVID bioinformatics resources. Nat. Protoc..

[25] Atai N.A., Balaj L., Van Veen H., Breakefield X.O., Jarzyna P.A., Van Noorden C.J.F., Skog J., Maguire C.A. (2013). Heparin blocks transfer of extracellular vesicles between donor and recipient cells. J. Neuro-Oncol..

[26] Ortega-Ribera M., Fernández-Iglesias A., Illa X., Moya A., Molina V., Maeso-Díaz R., Fondevila C., Peralta C., Bosch J., Villa R. (2018). Resemblance of the human liver sinusoid in a fluidic device with biomedical and pharmaceutical applications. Biotechnol. Bioeng..

[27] Friedman S.L. (2008). Hepatic Stellate Cells: Protean, multifunctional, and enigmatic cells of the liver. Physiol. Rev..

[28] Krenkel O., Tacke F. (2017). Liver macrophages in tissue homeostasis and disease. Nat. Rev. Immunol..

[29] Wang R., Ding Q., Yaqoob U., de Assuncao T.M., Verma V., Hirsova P., Cao S., Mukhopadhyay D., Huebert R.C., Shah V.H. (2015). Exosome Adherence and internalization by hepatic stellate cells triggers sphingosine 1-phosphate-dependent migration. J. Biol. Chem..

[30] Nojima H., Freeman C.M., Schuster R.M., Japtok L., Kleuser B., Edwards M.J., Gulbins E., Lentsch A.B. (2016). Hepatocyte exosomes mediate liver repair and regeneration via sphingosine-1-phosphate. J. Hepatol..

[31] Mullard A. (2020). IRAK4 degrader to take on innate immunity. Nat. Biotechnol..

[32] Tyanova S., Temu T., Sinitcyn P., Carlson A., Hein M.Y., Geiger T., Mann M., Cox J. (2016). The Perseus computational platform for comprehensive analysis of (prote)omics data. Nat. Methods.

[33] Huang H., Evankovich J., Yan W., Nace G., Zhang L., Ross M., Liao X., Billiar T., Xu J., Esmon C.T. (2011). Endogenous histones function as alarmins in sterile inflammatory liver injury through Toll-like receptor 9 in mice. Hepatology.

[34] 3Doñate F., McCrae K., Shaw D., Mazar A. (2004). Extracellular Tropomyosin: A novel common pathway target for anti-angiogenic therapy. Curr. Cancer Drug Targets.

[35] Bruix J., Reig M., Sherman M. (2016). Evidence-based diagnosis, staging, and treatment of patients with hepatocellular carcinoma. Gastroenterology.

[36] Shetty S., Lalor P.F., Adams D.H. (2018). Liver sinusoidal endothelial cells — gatekeepers of hepatic immunity. Nat. Rev. Gastroenterol. Hepatol..

[37] Gracia-Sancho J., Caparrós E., Fernández-Iglesias A., Francés R. (2021). Role of liver sinusoidal endothelial cells in liver diseases. Nat. Rev. Gastroenterol. Hepatol..

[38] Marrone G., Russo L., Rosado E., Hide D., García-Cardeña G., García-Pagán J.C., Bosch J., Gracia-Sancho J. (2013). The transcription factor KLF2 mediates hepatic endothelial protection and paracrine endothelial–stellate cell deactivation induced by statins. J. Hepatol..

[39] Winkler M., Staniczek T., Kürschner S.W., Schmid C.D., Schönhaber H., Cordero J., Kessler L., Mathes A., Sticht C., Neßling M. (2021). Endothelial GATA4 controls liver fibrosis and regeneration by preventing a pathogenic switch in angiocrine signaling. J. Hepatol..

[40] MacParland S.A., Liu J.C., Ma X.-Z., Innes B.T., Bartczak A.M., Gage B.K., Manuel J., Khuu N., Echeverri J., Linares I. (2018). Single cell RNA sequencing of human liver reveals distinct intrahepatic macrophage populations. Nat. Commun..

[41] Ramachandran P., Dobie R., Wilson-Kanamori J.R., Dora E.F., Henderson B.E.P., Luu N.T., Portman J.R., Matchett K.P., Brice M., Marwick J.A. (2019). Resolving the fibrotic niche of human liver cirrhosis at single-cell level. Nat. Cell Biol..

[42] Su T., Yang Y., Lai S., Jeong J., Jung Y., McConnell M., Utsumi T., Iwakiri Y. (2021). Single-cell transcriptomics reveals zone-specific alterations of liver sinusoidal endothelial cells in cirrhosis. Cell. Mol. Gastroenterol. Hepatol..

[43] Momen-Heravi F., Bala S., Kodys K., Szabo G. (2015). Exosomes derived from alcohol-treated hepatocytes horizontally transfer liver specific miRNA-122 and sensitize monocytes to LPS. Sci. Rep..

[44] Otogawa K., Ogawa T., Shiga R., Ikeda K., Kawada N. (2008). Induction of tropomyosin during hepatic stellate cell activation and the progression of liver fibrosis. Hepatol. Int..

[45] Mölleken C., Sitek B., Henkel C., Poschmann G., Sipos B., Wiese S., Warscheid B., Broelsch C., Reiser M., Friedman S.L. (2008). Detection of novel biomarkers of liver cirrhosis by proteomic analysis. Hepatology.

[46] Bhandari S., Li R., Simón-Santamaría J., McCourt P., Johansen S.D., Smedsrød B., Martinez-Zubiaurre I., Sørensen K.K. (2020). Transcriptome and proteome profiling reveal complementary scavenger and immune features of rat liver sinusoidal endothelial cells and liver macrophages. BMC Mol. Cell Biol..

[47] Ganesan L.P., Kim J., Wu Y., Mohanty S., Phillips G.S., Birmingham D.J., Robinson J.M., Anderson C.L. (2012). FcγRIIb on liver sinusoidal endothelium clears small immune complexes. J. Immunol..

