# Supplementary material for: Transcriptomic Profiling of the Liver Sinusoidal Endothelium during Cirrhosis Reveals Stage-Specific Secretory Signature
Source: Cancers (Basel). 2021 May 29;13(11):2688. doi: 10.3390/cancers13112688 (PMC8198220; doi:10.3390/cancers13112688)
Supplement: Supplementary file 1 [file cancers-13-02688-s001.zip › cancers-1211497-supplementary.pdf]

cancers

MDPI

Supplementary Materials: Transcriptomic Profiling of the Liver Sinusoidal Endothelium During Cirrhosis Reveals Stage-Specific Secretory Signature

Nicolò Manicardi, Anabel Fernández-Iglesias, Laia Abad-Jordà, Felix Royo, Mikel Azkargorta, Martí Ortega-Ribera, David Sanfeliu-Redondo, Ana Martínez-Alcocer, Felix Elortza, Amelia J Hessheimer, Constantino Fondevila, Juan José Lozano, Juan Carlos García-Pagán, Jaime Bosch, Francisco Javier Cubero, Agustín Albillos, Javier Vaquero, Juan M. Falcón-Pérez and Jordi Gracia-Sancho

Citation: Manicardi, N.; Fernández-Iglesias, A.; Abad-Jordà, L.; Royo, F.; Azkargorta, M.; Ortega-Ribera, M.; Sanfeliu-Redondo, D.; Martínez-Alcocer, A.; Elortza, F.; Hessheimer, A.J.; et al. Transcriptomic Profiling of The Liver Sinusoidal Endothelium During Cirrhosis Reveals Stage-Specific Secretory Signature. *Cancers* **2021**, *13*, 2688. https://doi.org/10.3390/cancers13112688

Academic Editor: Claudio Tiribelli

Received: 21 April 2021
Accepted: 25 May 2021
Published: 29 May 2021

Publisher's Note: MDPI stays neutral with regard to jurisdictional claims in published maps and institutional affiliations.

Copyright: © 2021 by the authors. Licensee MDPI, Basel, Switzerland. This article is an open access article distributed under the terms and conditions of the Creative Commons Attribution (CC BY) license (http://creativecommons.org/licenses/by/4.0/).

Supplementary Materials & Methods:

1. Rat Hepatic Cells Isolation

Rats weighing 300–350 g were anesthetized with ketamine (100 mg/kg body weight, Imalgene 1000; Merial) plus midazolam (5 mg/kg body weight; Laboratorio Reig Jofre, S.A., ES) administered intraperitoneally.

Hepatocytes and NPCs were isolated using a well-established protocol as mentioned in the main manuscript. Briefly, rat livers were perfused through the portal vein with Hanks hepes buffer containing ethylene glycol-bis (2-aminoethylether)-N,N,N′,N′-tetraacetic acid (E4378, Sigma, USA) and BSA (A1391,0100, Applichem, DE). Liver digestion was performed during 30 min with Hanks hepes containing 0.015% collagenase A (10103586001, Roche, DE). The resultant digested liver was excised, and additional *in vitro* digestion was performed at 37°C with 0.01% collagenase A for 10 min. Disaggregated tissue was filtered using 100 μm nylon strainer, collected in cold Krebs buffer and centrifuged at 50g for 5 min at 4ºC for hepatocytes purification. Hepatocytes were seeded in collagen coated 48 wells plate and cultured in DMEMF12 (21041-025, Gibco, UK) supplemented with 2% exosome-depleted FBS (A2720803, Gibco, UK), 1% L-glutamine, 1% penicillin plus 1% streptomycin, 1 nM dexamethasone (D4902, Sigma, USA), 1 μM insulin (103755, HCB) and 1% amphotericin B.

The resulting supernatant was centrifuged twice at 800g for 10 min and the obtained pellet was subjected to differential centrifugation (1400g, 21 minutes at 4°C without break) using iodixanol (D1556, Sigma, MO, USA) to perform two gradients (17-11.5%). The upper interphase of the gradient contained HSCs while the lower interphase was enriched of HMΦ and LSECs. HSCs were collected, seeded in 48 wells plated and cultured in IMDM (21980-032, Invitrogen, Gibco, UK) supplemented with 10% exosome-depleted FBS, 1% L-glutamine, 1% penicillin plus 1% streptomycin and 1% amphotericin B.

HMΦ and LSECs were collected and LSECs were isolated by selective cell adhesion to substrate. HMΦ were seeded in 48 wells plate and cultured in RPMI-1640 medium supplemented with 10% exosome-depleted FBS, 1% L-glutamine, 1% penicillin plus 1% streptomycin and 1% amphotericin B.

LSECs were seeded on collagen coated plates (37°C, 5% CO_2) in RPMI-1640 without Phenol Red (01-103-1A, Biological Industries, CT, USA) supplemented with penicillin-streptomycin solution 1%, amphotericin B 1%, L-glutamine 1% and exosome-depleted FBS 10%. After 1h, LSECs were lysed with QIAzol reagent for total RNA extraction using the miRNeasy microkit following the manufacturer's instructions. RNA samples were stored at -80°C for subsequent total RNA sequencing. In additional experiments, LSECs were cultured 16h for supernatant collection.

2. Human Liver Sinusoidal Endothelial Cells isolation

Liver tissue piece approximately weighting 10–20g was cannulated and perfused with Hanks hepes (H3375, Sigma, USA) buffer to eliminate the excessive blood [1].

Following, digestion process was performed with Hanks hepes containing 280 U/mL of collagenase A (C5138, Sigma, Spain) and DNase (10 mg/mL) (18535, Serva, Germany) for 20 min with recirculation buffer. The resultant digested liver was removed, disaggregated, filtered using sterile gauze, and collected in cold Krebs buffer to discard hepatocytes by low-speed centrifugation. Non-parenchymal cells (NPCs) were pelleted by centrifugation at 800g for 10 minutes at 4ºC, and LSECs were labeled by incubation with human CD32 primary antibody (1:1000; Fitzgerald, MA, USA) and isolated by immunomagnetism with Dynabeads® CELLection Pan Mouse IgG Kit (11531D, ThermoFisher Scientific, USA) according to the manufacturer's instructions [2].

CD32b was selected as LSECs phenotypic marker considering its abundance and characteristic expression in healthy and cirrhotic LSECs [2–5], which indeed agreed with CD32b protein expression in whole human liver tissue assessed by IF (Figure S6A). Additionally, the gene set enrichment analysis (GSEA) comparing the human CD32b$^+$ cells used in the present study and different subsets of endothelial cells described in [5] suggest that isolated human CD32b$^+$ cells mostly correspond sub-population Endo(1), identified as LSECs, Endo(3) and Endo(6), identified as scar-associated LSECs, in the single-cell analysis of *Ramachandran P* et al. [5] (Figure S6C-D).

LSECs were resuspended in RPMI-1640 (010101-1A, Biological Industries, CT, USA) supplemented with 1% FBS (04-077-1A, Biological Industries, CT, USA), 1% penicillin and 1% streptomycin (03-331-1C, Biological Industries, CT, USA), 2 mM L-glutamine (25030-024, Gibco, UK), 1% amphotericin B (03-029-1C, Biological Industries, CT, USA), 1% endothelial cell growth supplement (ECGS, 02-102, Merck, DE) and 100 mg/mL heparin (H3393-100KU, Sigma, MO, USA), and seeded in collagen-coated plates for 1 h (37°C, 5% CO_2). LSECs were collected with QIAzol lysis reagent (79306, Qiagen, MA, USA) for RNA extraction using miRNeasy microkit (74004, Qiagen, DE) for subsequent RNA sequencing. Purity of isolated LSECs was routinely validated by presence of fenestrae visualized by scanning electron microscopy (Figure S6B), and very low or non-detectable expression of HMΦs, HSCs and hepatocytes markers (Figure S6G).

3. Nanoparticle Tracking Analysis

Quantity and concentration of isolated EVs were assessed by nanoparticle tracking analysis using the NanoSight NS300 instrumentation (Malvern Panalytical, UK). EVs samples were diluted with PBS and 3 videos of 20 seconds were taken for each sample. Results were analyzed using NanoSight software NTA 3.4.

4. MicroBCA™ Colorimetric Protein Assay

The total amount of EVs-associated protein of each secretome fraction was quantified by MicroBCA colorimetric protein assay (23235, Thermo Scientific, IL, USA) following the manufacturer's instructions.

5. EVs Immunoblotting

EVs purity was assessed by immuno-blot detection of EVs associated markers (CD63, CD81) and endoplasmic reticulum and mitochondria related ones (GRP-78, COX-IV). As suggested by the guidelines [6], samples for CD63 and CD81 detection were prepared in denaturing but not reducing conditions while denaturing and reducing conditions were used for GRP-78 and COX-IV detection. For detailed description of the antibodies see Table S17.

6. Cryo-Electron Microscopy

Small EVs samples were analysed by cryo-electron microscopy in collaboration with the Electron Microscopy Platform CIC BioGUNE (Derio, Spain). EVs preparations were directly adsorbed onto glow-discharged holey carbon grids (QUANTIFOIL, Germany). Grids were blotted at 95% humidity and rapidly plunged into liquid ethane with the aid

of a VITROBOT (Maastricht Instruments BV, The Netherlands). Vitrified samples were imaged at liquid nitrogen temperature using a JEM-2200FS/CR transmission cryo-electron microscope (JEOL, Japan) equipped with a field emission gun and operated at an acceleration voltage of 200 kV. Cryo-TEM procedure spotted a heterogeneous population of LSECs EVs (Figure S4C).

7. LSECs' Secretome Proteomics

7.1. In solution digestion:

Samples were incubated in a solution containing 7M urea 2M Thiourea 4% CHAPS and 5mM DTT for 30 min at RT under agitation and digested following the filter-aided FASP protocol described by Wisniewski et al [7] with minor modifications. Trypsin was added to a trypsin:protein ratio of 1:50, and the mixture was incubated overnight at 37ºC, dried out in a RVC2 25 speedvac concentrator (Christ), and resuspended in 0.1% FA. Peptides were desalted and resuspended in 0.1% FA using C18 stage tips (Millipore).

7.2. Mass spectrometry analysis:

Samples were analyzed in a novel hybrid trapped ion mobility spectrometry – quadrupole time of flight mass spectrometer (timsTOF Pro with PASEF, Bruker Daltonics) coupled online to a nanoElute liquid chromatograph (Bruker). This mass spectrometer takes advantage of a novel scan mode termed parallel accumulation – serial fragmentation (PASEF), which multiplies the sequencing speed without any loss in sensitivity [8] and has been proven to provide outstanding analytical speed and sensibility for proteomics analyses [9]. Sample (200ng) was directly loaded in a 15 cm Bruker nanoelute FIFTEEN C18 analytical column (Bruker) and resolved at 400 nL/min with a 30 min gradient. Column was heated to 50ºC using an oven.

Protein identification and quantification was carried out using PEAKS software (Bioinformatics solutions). Searches were carried out against a database consisting of *Bos taurus* and *Rattus norvegicus* entries (Uniprot/Swissprot), with precursor and fragment tolerances of 20 ppm and 0.05 Da. Only proteins identified with at least two peptides at FDR<1% were considered for further analysis. Data was loaded onto Perseus platform and further processed (log2 transformation, imputation). A t-test was applied in order to determine the statistical significance of the differences detected and heatmaps were generated. Results were then filtered for *Rattus norvegicus*.

8. Selection Criteria for Small Evs Proteomics

The whole proteome sequencing generated 1643 results that were subsequently filtered with the following criteria. The first criterium was to consider those detections in which more than 1 peptide had been detected for each protein, reducing the list to 1042 detections. Of the 1042 detections, 381 were for *Bos Taurus* and 661 for *Rattus norvegicus*. The following criterium was to separate the entries for species, considering only the 661 proteins detected for *Rattus norvegicus*. We then observed whether the proteins were also been detected in the medium proceeding from culture wells where no cells had been seeded. The medium was supplemented as detailed in the paragraph 1 of the present document. With this third criterium, 428 proteins were undetected in the medium while 233 did. From the remaining 428 proteins we decided to further sieve them with and additional criterium, considering only those proteins whose Unique/Peptide ratio were > 0.5. With this one last criterium we wanted to be sure that the peptide sequences detected by the MS-LC were unique for the protein associated to those peptides. The remaining proteins were thus narrowed down to the 345 that had been used to run the IPA analysis.

9. HSCs Immunofluorescence

Cells were seeded on coverglasses of 18 mm diameter (Cat. #72222-01 Circular coverglass, Electron Microscopy Sciences, USA) and treated for 48h with 500 ng/μL complete

secretome (CS) proceeding from healthy and cirrhotic rat LSECs. Cells were fixed with 4% paraformaldehyde solution in PBS (SC-281692, Santa Cruz Biotechnology, CA, USA) for 10 minutes, rinsed thrice with PBS and permeabilized with 0.1% triton X-100 (9002-93-1, Sigma, MO, USA) in PBS for 5 minutes. Unspecific binding sites were blocked for 30 minutes with 1% BSA in PBS and subsequently incubated 2h at RT with the primary antibody against αSMA (1:200, 2547 Sigma). Anti-mouse Alexa Fluor 488 (1:300, A21202, Invitrogen, OR, USA) was incubated 1h at RT in combination with DAPI to stain nuclei (1 μg/mL, 10232676001, Roche, DE). Fifteen images per preparation were captured with a spectral confocal microscope (Leica TCS-SP5, DE). Images were taken at 400X magnification and image analysis of fluorescence was performed using ImageJ (Fiji) software [10]. The same settings were maintained while quantifying images from the same experiment. Intensities were normalized to the number of cells per field.

10. cDNA conversion and real time RT-PCR assay

Equal amount of RNA was converted to cDNA using the High Capacity cDNA Reverse Transcription kit (4368814, Applied Biosystems™, Vilnius, LT). cDNA samples were amplified by real-time RT-PCR on ABI PRISM 7900HT (Applied Biosystems™). Rat specific primers designed using sequence data and Nucleotide BLAST software from the National Center for Biotechnology Information (NCBI, MD, USA) database, and Taqman predesigned probes for TPM1 experiments were used with specific mastermix (Applied Biosystems™) (Table S18). Gene expression of specific gene was calculated based on the ΔΔCt method. The results were expressed as $2^{-\Delta\Delta Ct}$ referred as fold-expression compared to control group.

11. Western Blot Assay

Cells samples were collected with protein lysis buffer Halt™ Protease and Phosphatase Inhibitor Cocktail (78448, Thermo Fisher Scientific, USA), centrifuged at 4°C 10 min at 14,000g and supernatant was mixed with Laemmli sample buffer (1610747, Bio-Rad Laboratories, CA, USA) and β-mercaptoethanol following manufacturer's instructions. Equal amounts of protein were run on sodium dodecylsulphate polyacrylamide gel. Transferred nitrocellulose membrane was blocked with Tris-buffer saline containing 0.05% Tween-20 and 5% non-fat dry milk or 3% BSA, and subsequently incubated overnight at 4ºC with primary antibodies against TPM1 and α-Tubulin as endogenous control; all at 1:1000 dilution. After the incubation with the corresponding secondary peroxidase-coupled antibody, blots were revealed by chemiluminescence (ECL, Amersham) using LAS4000 (GE Healthcare, Spain) and protein expression was evaluated by densitometric analysis using Image Studio software (LI-COR Biosciences, USA). Whole membrane blots are represented in Figure S7. For detailed description of the antibodies see Table S17.

Supplementary Tables

Table S1. Top 25 up- & down-regulated genes in CCl_4-cirrhotic rat LSECs.

Gene symbol	Fold-change	p-value	fdr
Ccl21	335.00	0.000	0.000
Sema3a	63.56	0.000	0.000
Piezo2	33.79	0.000	0.000
Clic5	26.83	0.000	0.000
Rgs5	26.12	0.000	0.000
Igsf3	25.93	0.000	0.000
Sema3d	25.04	0.000	0.000
Chrdl1	24.32	0.000	0.000
Flnc	22.28	0.000	0.000
Mmp12	20.30	0.000	0.000

Cyp1a1	19.48	0.000	0.002
Lpl	19.44	0.000	0.000
Casc4	19.24	0.000	0.000
Dcn	19.24	0.000	0.000
Il6	19.12	0.000	0.000
Robo2	19.07	0.000	0.000
Igsf10	18.95	0.000	0.000
Ano1	18.36	0.000	0.000
Pnoc	17.98	0.000	0.000
Pdpn	17.93	0.000	0.000
Prss23	17.82	0.000	0.000
Adamts12	16.87	0.000	0.000
Ogn	16.56	0.000	0.000
Mgp	16.06	0.000	0.000
Ednrb	15.82	0.000	0.000
Gcat	-5.04	0.000	0.001
Dcxr	-5.10	0.000	0.002
Gpr153	-5.13	0.000	0.000
Plch2	-5.23	0.001	0.004
Akr7a2	-5.28	0.000	0.000
Hoxb5	-5.31	0.000	0.000
Cpne5	-5.33	0.000	0.000
Igdcc3	-5.33	0.001	0.004
Kcp	-5.43	0.000	0.000
Slco2b1	-5.48	0.000	0.000
Slc22a8	-5.67	0.006	0.013
Gpt	-6.35	0.001	0.003
Tmem132e	-6.61	0.000	0.000
Jph3	-6.62	0.000	0.000
Slc46a1	-6.72	0.000	0.001
Cib2	-6.93	0.000	0.000
Ptpru	-7.48	0.000	0.000
Hal	-8.02	0.002	0.005
Cabp2	-8.22	0.001	0.003
Igf2bp2	-8.89	0.000	0.000
Hba1	-9.48	0.034	0.055
Cntfr	-9.54	0.000	0.000
Stac3	-16.10	0.001	0.003
Grik4	-18.73	0.000	0.000
Ihh	-31.75	0.000	0.000

Table S2. Top 25 up- & down-regulated genes in TAA-cirrhotic rat LSECs.

Gene symbol	Fold-change	p-value	fdr
Ccl21	279.90	0.000	0.000
Chrdl1	215.06	0.000	0.000
Pdpn	93.61	0.000	0.000
Igfbp5	92.49	0.000	0.000
Adamts8	76.68	0.000	0.000
Flnc	69.78	0.000	0.000
Sema3a	69.32	0.000	0.000
Igsf3	57.78	0.000	0.000

Piezo2	46.72	0.000	0.000
Loxl1	40.65	0.000	0.000
Pnoc	39.43	0.000	0.000
Notch3	37.98	0.000	0.000
Col12a1	36.61	0.000	0.000
Cdh3	36.02	0.000	0.000
Fblim1	34.46	0.000	0.000
Pdgfrb	34.18	0.000	0.000
Ednra	32.14	0.000	0.000
Prss23	31.34	0.000	0.000
Lamb1	30.41	0.000	0.000
Plod2	30.15	0.000	0.000
Adamts2	29.52	0.000	0.000
Col1a1	29.06	0.000	0.000
Col8a1	27.94	0.000	0.000
Ltbp2	27.69	0.000	0.000
Cxcl14	27.49	0.000	0.000
Ikzf3	-11.51	0.000	0.002
LOC100359515	-11.76	0.003	0.009
Il2rb	-12.69	0.000	0.001
Tagap	-12.94	0.000	0.001
Klrc2	-13.27	0.000	0.001
Cd7	-13.67	0.000	0.001
Zbp1	-13.80	0.000	0.001
Klrb1b	-15.57	0.000	0.001
Art2b	-16.00	0.000	0.000
Klrd1	-16.55	0.000	0.000
Mylk3	-16.79	0.000	0.000
Cntfr	-17.20	0.000	0.000
Asb2	-18.88	0.000	0.000
LOC259244	-18.89	0.000	0.002
Cd69	-18.91	0.000	0.001
Ccl5	-19.09	0.000	0.000
Nkg7	-19.92	0.000	0.001
LOC259245	-21.38	0.000	0.002
Sh2d2a	-23.31	0.000	0.001
Igf2bp2	-27.44	0.000	0.000
Klri1	-27.79	0.000	0.001
Ihh	-33.16	0.000	0.000
Igdcc3	-33.90	0.000	0.000
Xcl1	-44.04	0.000	0.000
Oas2	-45.28	0.000	0.000

Table S3. Top 25 up- & down-regulated genes in cBDL-cirrhotic rat LSECs.

Gene symbol	Fold-change	p-value	fdr
Mmp12	126.34	0.000	0.000
Gpnmb	78.44	0.000	0.000
Igfbp5	59.80	0.000	0.000
Ccl21	57.11	0.000	0.002
Fblim1	56.00	0.000	0.000
Igsf3	46.96	0.000	0.000

Ms4a4a	44.65	0.000	0.002
Pla2g7	44.07	0.000	0.000
Cd300lb	40.98	0.001	0.002
Nat8l	40.21	0.000	0.000
Chrdl1	40.12	0.000	0.000
Mcemp1	35.02	0.000	0.000
Lpl	34.36	0.000	0.000
Pnoc	33.40	0.000	0.003
Klhl41	33.20	0.000	0.000
Lgals3	31.47	0.000	0.001
B3galnt1	31.25	0.000	0.000
Abcc9	30.32	0.000	0.000
Lgals1	30.31	0.000	0.000
Piezo2	29.86	0.000	0.003
Capg	29.45	0.000	0.001
Clec10a	29.40	0.000	0.002
Sh3pxd2b	28.73	0.000	0.000
Rab7b	28.48	0.000	0.001
Fn1	28.32	0.000	0.000
Cntfr	-12.61	0.000	0.000
Apoc2	-12.80	0.001	0.004
Hal	-13.07	0.002	0.007
Alb	-13.27	0.000	0.000
Tmem132e	-13.32	0.000	0.000
Slco1a1	-14.34	0.000	0.003
Serpina6	-14.36	0.000	0.001
Fabp1	-15.86	0.001	0.006
Vwa3b	-15.91	0.000	0.000
Col4a3	-15.92	0.000	0.000
Cyp4a2	-16.48	0.000	0.001
Cyp26c1	-16.60	0.000	0.000
Cyp2c7	-17.08	0.001	0.005
Astn2	-17.61	0.000	0.000
Cabp2	-23.26	0.001	0.003
Cyp26a1	-23.40	0.000	0.000
RGD1307603	-35.60	0.000	0.001
Kcnd3	-38.19	0.000	0.000
Apoa4	-38.44	0.000	0.001
Slc22a8	-39.52	0.000	0.001
Stac3	-52.67	0.000	0.000
Mylk3	-55.39	0.000	0.000
Ihh	-59.76	0.000	0.000
Igdcc3	-70.70	0.000	0.000
Car3	-865.45	0.000	0.000

Table S4. Top common up- and down-regulated genes between CCl_4-, TAA- and cBDL cirrhotic rat LSECs.

Gene symbol	Fold-change			p-value			fdr		
	CCl_4	TAA	cBDL	CCl_4	TAA	cBDL	CCl_4	TAA	cBDL
Ccl21	335.00	279.90	57.11	0.000	0.000	0.000	0.000	0.000	0.002
Sema3a	63.56	69.32	26.82	0.000	0.000	0.000	0.000	0.000	0.001

Piezo2	33.79	46.72	29.86	0.000	0.000	0.000	0.000	0.000	0.003
Clic5	26.83	9.31	12.50	0.000	0.001	0.000	0.000	0.004	0.000
Rgs5	26.12	19.96	17.96	0.000	0.000	0.003	0.000	0.000	0.008
Igsf3	25.93	57.78	46.96	0.000	0.000	0.000	0.000	0.000	0.000
Sema3d	25.04	13.45	7.46	0.000	0.000	0.010	0.000	0.000	0.023
Chrdl1	24.32	215.06	40.12	0.000	0.000	0.000	0.000	0.000	0.000
Flnc	22.28	69.78	11.93	0.000	0.000	0.001	0.000	0.000	0.003
Cyp1a1	19.48	22.34	10.25	0.000	0.000	0.017	0.002	0.001	0.036
Lpl	19.44	20.73	34.36	0.000	0.000	0.000	0.000	0.000	0.000
Casc4	19.24	8.41	3.43	0.000	0.000	0.050	0.000	0.000	0.086
Dcn	19.24	11.13	23.09	0.000	0.000	0.001	0.000	0.000	0.003
Il6	19.12	18.28	4.46	0.000	0.000	0.005	0.000	0.000	0.014
Igsf10	18.95	22.59	10.58	0.000	0.000	0.002	0.000	0.000	0.006
Ano1	18.36	16.34	7.21	0.000	0.000	0.006	0.000	0.000	0.015
Pnoc	17.98	39.43	33.40	0.000	0.000	0.001	0.000	0.000	0.003
Pdpn	17.93	93.61	19.98	0.000	0.000	0.000	0.000	0.000	0.000
Prss23	17.82	31.34	8.76	0.000	0.000	0.003	0.000	0.000	0.010
Adamts12	16.87	15.53	8.58	0.000	0.000	0.000	0.000	0.000	0.001
Mgp	16.06	19.08	21.59	0.000	0.000	0.005	0.000	0.000	0.013
Ednrb	15.82	23.53	12.24	0.000	0.000	0.000	0.000	0.000	0.000
Apba2	15.81	5.95	3.25	0.000	0.000	0.015	0.000	0.001	0.032
Plod2	15.26	30.15	26.67	0.000	0.000	0.000	0.000	0.000	0.000
Col12a1	14.73	36.61	25.48	0.000	0.000	0.001	0.000	0.000	0.005
Plpp7	-4.37	-2.98	-5.17	0.000	0.002	0.000	0.000	0.006	0.000
Fam78b	-4.62	-3.28	-3.07	0.000	0.000	0.000	0.000	0.000	0.000
Astn2	-4.65	-2.23	-17.61	0.000	0.000	0.000	0.000	0.000	0.000
Gstp1	-4.70	-1.56	-2.56	0.000	0.000	0.000	0.000	0.001	0.000
Aatk	-4.76	-2.62	-4.82	0.000	0.000	0.000	0.000	0.000	0.000
Ier5l	-4.80	-1.79	-1.84	0.000	0.002	0.006	0.000	0.008	0.015
Lrg1	-4.84	-2.40	-4.62	0.000	0.000	0.000	0.000	0.000	0.000
RT1-S2	-4.87	-2.20	-2.34	0.005	0.000	0.000	0.011	0.000	0.002
Islr	-5.00	-1.74	-2.34	0.000	0.028	0.021	0.000	0.056	0.044
Gpr153	-5.13	-1.72	-4.90	0.000	0.000	0.000	0.000	0.002	0.000
Hoxb5	-5.31	-2.76	-3.89	0.000	0.000	0.000	0.000	0.001	0.000
Igdcc3	-5.33	-33.90	-70.70	0.001	0.000	0.000	0.004	0.000	0.000
Kcp	-5.43	-4.47	-10.80	0.000	0.000	0.000	0.000	0.002	0.000
Slco2b1	-5.48	-4.24	-9.93	0.000	0.000	0.000	0.000	0.000	0.002
Tmem132e	-6.61	-2.56	-13.32	0.000	0.000	0.000	0.000	0.001	0.000
Jph3	-6.62	-2.27	-10.63	0.000	0.000	0.000	0.000	0.002	0.000
Cib2	-6.93	-2.01	-3.54	0.000	0.006	0.016	0.000	0.015	0.035
Ptpru	-7.48	-2.41	-11.87	0.000	0.000	0.000	0.000	0.000	0.001
RGD1307603	-7.81	-2.52	-35.60	0.003	0.019	0.000	0.007	0.041	0.001
Cabp2	-8.22	-4.53	-23.26	0.001	0.001	0.001	0.003	0.003	0.003
Igf2bp2	-8.89	-27.44	-7.25	0.000	0.000	0.000	0.000	0.000	0.000
Cntfr	-9.54	-17.20	-12.61	0.000	0.000	0.000	0.000	0.000	0.000
Stac3	-16.10	-4.71	-52.67	0.001	0.000	0.000	0.003	0.000	0.000
Grik4	-18.73	-5.97	-7.94	0.000	0.000	0.002	0.000	0.000	0.007
Ihh	-31.75	-33.16	-59.76	0.000	0.000	0.000	0.000	0.000	0.000

Table S5. Common DEG between CCl_4-, TAA- and cBDL-cirrhotic rat LSECs defining extracellular exosomes components.

Cellular Component: extracellular exosomes (GO:0070062)	
**Gene symbol**	**Gene Name**
Nt5e	5' nucleotidase, ecto
LOC108351936	60S ribosomal protein L30-like
Aebp1	AE binding protein 1
Ahnak	AHNAK nucleoprotein
Cd9	CD9 molecule
Efhd1	EF-hand domain family, member D1
Efemp2	EGF-containing fibulin-like extracellular matrix protein 2
Ephb4	EPH receptor B4
Fkbp5	FK506 binding protein 5
Gprc5b	G protein-coupled receptor, class C, group 5, member B
Glipr2	GLI pathogenesis-related 2
Hid1	HID1 domain containing
RT1-S3	RT1 class Ib, locus S3
Arhgef18	Rho/Rac guanine nucleotide exchange factor 18
S100a10	S100 calcium binding protein A10
Sh3bgrl	SH3 domain binding glutamate-rich protein like
Slc9a3r1	SLC9A3 regulator 1
Steap4	STEAP4 metalloreductase
Timp2	TIMP metallopeptidase inhibitor 2
Acta2	actin, alpha 2, smooth muscle, aorta
Actn1	actinin, alpha 1
Angpt1	angiopoietin 1
Anxa1	annexin A1
Anxa2	annexin A2
Ano1	anoctamin 1
Antxr1	anthrax toxin receptor 1
Apoa2	apolipoprotein A2
Apod	apolipoprotein D
Apoe	apolipoprotein E
Bgn	biglycan
Bphl	biphenyl hydrolase like
Creb5	cAMP responsive element binding protein 5
Cactin	cactin, spliceosome C complex subunit
Cdh13	cadherin 13
Cdh2	cadherin 2
Car2	carbonic anhydrase 2
Cpz	carboxypeptidase Z
Chmp4c	charged multivesicular body protein 4C
Clic5	chloride intracellular channel 5
F3	coagulation factor III, tissue factor
Col1a2	collagen type I alpha 2 chain
Col5a1	collagen type V alpha 1 chain
Col5a3	collagen type V alpha 3 chain
Col6a1	collagen type VI alpha 1 chain
Col6a2	collagen type VI alpha 2 chain
Col8a1	collagen type VIII alpha 1 chain
Col12a1	collagen type XII alpha 1 chain

Cfb	complement factor B
Cfh	complement factor H
Cryab	crystallin, alpha B
Crispld2	cysteine-rich secretory protein LCCL domain containing 2
Dsg2	desmoglein 2
Dnm3	dynamin 3
Entpd1	ectonucleoside triphosphate diphosphohydrolase 1
Enpp3	ectonucleotide pyrophosphatase/phosphodiesterase 3
Epcam	epithelial cell adhesion molecule
Eef1a1	eukaryotic translation elongation factor 1 alpha 1
Fabp3	fatty acid binding protein 3
Fabp4	fatty acid binding protein 4
Fbn1	fibrillin 1
Fbln1	fibulin 1
Fbln5	fibulin 5
Lgals9	galectin 9
Gja1	gap junction protein, alpha 1
Gfpt1	glutamine fructose-6-phosphate transaminase 1
Gstp1	glutathione S-transferase pi 1
Gpx3	glutathione peroxidase 3
Gpc1	glypican 1
Golm1	golgi membrane protein 1
Gas6	growth arrest specific 6
Hist1h2ao	histone cluster 1, H2ao
Hist2h4	histone cluster 2, H4
Islr	immunoglobulin superfamily containing leucine-rich repeat
Insr	insulin receptor
Igf2r	insulin-like growth factor 2 receptor
Igf2	insulin-like growth factor 2
Itgav	integrin subunit alpha V
Knl1	kinetochore scaffold 1
Lamb1	laminin subunit beta 1
Ltbp2	latent transforming growth factor beta binding protein 2
Lrg1	leucine-rich alpha-2-glycoprotein 1
Lrrk2	leucine-rich repeat kinase 2
Lcn2	lipocalin 2
Lpl	lipoprotein lipase
Ly75	lymphocyte antigen 75
Mgat5	Mannosyl (alpha-1,6-)-glycoprotein beta-1,6-N-acetyl-glucosaminyl-transferase
Mgp	matrix Gla protein
Mfge8	milk fat globule-EGF factor 8 protein
Mnda	myeloid cell nuclear differentiation antigen
Myof	myoferlin
Myo5a	myosin VA
Nebl	nebulette
Nid2	nidogen 2
Olr1	oxidized low density lipoprotein (lectin-like) receptor 1
Ppic	peptidylprolyl isomerase C
Ppl	periplakin
Plat	plasminogen activator, tissue type

Pdgfrb	platelet derived growth factor receptor beta
Plekha7	pleckstrin homology domain containing A7
Plxdc2	plexin domain containing 2
Pabpc1	polyA binding protein, cytoplasmic 1
Pcbp3	polyrC binding protein 3
Prnp	prion protein
Plod2	procollagen lysine, 2-oxoglutarate 5-dioxygenase 2
Prss23	protease, serine, 23
Prss8	protease, serine, 8
Prkar2b	protein kinase cAMP-dependent type 2 regulatory subunit beta
Ptpn13	protein tyrosine phosphatase, non-receptor type 13
Plp2	proteolipid protein 2
Rhoq	ras homolog family member Q
Rhot2	ras homolog family member T2
Rtn4rl1	reticulon 4 receptor-like 1
Sfrp1	secreted frizzled-related protein 1
Sema3c	semaphorin 3C
Sema3g	semaphorin 3G
Serpine1	serpin family E member 1
Serping1	serpin family G member 1
Serpini1	serpin family I member 1
Smim1	small integral membrane protein 1
Slc12a2	solute carrier family 12 member 2
Slc25a25	solute carrier family 25 member 25
Slc4a4	solute carrier family 4 member 4
Snx18	sorting nexin 18
Sat2	spermidine/spermine N1-acetyltransferase family member 2
Susd2	sushi domain containing 2
Syt7	synaptotagmin 7
Sdc4	syndecan 4
Txnrd1	thioredoxin reductase 1
Tars	threonyl-tRNA synthetase
Thbs1	thrombospondin 1
Thsd7a	thrombospondin type 1 domain containing 7A
Trim36	tripartite motif-containing 36
Wars	tryptophanyl-tRNA synthetase
Tinagl1	tubulointerstitial nephritis antigen-like 1
Ttyh3	tweety family member 3
Twsg1	twisted gastrulation BMP signaling modulator 1
Tpst2	tyrosylprotein sulfotransferase 2
Vamp5	vesicle-associated membrane protein 5
Vim	vimentin
Vwa1	von Willebrand factor A domain containing 1

Table S6. Top 25 up- & down-regulated genes in human cirrhotic CD32b+ LSECs.

Gene symbol	Fold-change	p-value	fdr
RHOD	30.72	0.005	0.070
RBM14-RBM4	25.84	0.002	0.056
NLRP6	20.85	0.002	0.056
RP11-727F15.14	19.78	0.001	0.046
FN1	19.18	0.005	0.072

RARRES2	17.40	0.006	0.074
MXRA8	17.33	0.000	0.037
RMRP	16.15	0.001	0.046
ISLR	14.89	0.001	0.046
AC010970.2	14.01	0.001	0.043
FCN1	13.86	0.000	0.037
RNVU1-18	13.62	0.008	0.079
RN7SL1	13.25	0.001	0.043
JSRP1	13.21	0.016	0.105
RPPH1	12.50	0.001	0.048
RN7SL2	12.31	0.002	0.050
SNORD3A	12.08	0.000	0.037
CFD	11.02	0.000	0.037
HK3	10.90	0.003	0.059
CH507-513H4.5	10.82	0.002	0.050
IGF2	10.69	0.000	0.037
IGFBP2	10.30	0.003	0.062
LTB	10.09	0.005	0.072
RN7SK	10.06	0.007	0.078
TYMP	9.88	0.000	0.037
MAPRE1	-33.80	0.009	0.084
CDK2AP1	-34.04	0.009	0.084
GPKOW	-34.55	0.007	0.079
NPL	-34.92	0.018	0.111
EHD4	-34.99	0.003	0.063
MBTPS1	-36.48	0.021	0.119
RDX	-37.46	0.005	0.071
MASP1	-40.57	0.004	0.069
UBE2G2	-41.71	0.001	0.047
ADI1	-45.06	0.007	0.078
ADGRL2	-45.88	0.019	0.115
PROCR	-46.41	0.000	0.037
MXRA7	-47.09	0.005	0.072
BTG1	-48.14	0.021	0.119
CLIC4	-48.63	0.001	0.046
ALDH2	-49.32	0.008	0.080
LGALS3	-49.53	0.004	0.069
TMEM165	-56.82	0.002	0.056
PRKAG1	-57.95	0.003	0.061
DCTN2	-61.63	0.009	0.083
COX7B	-66.25	0.005	0.071
PDCL	-67.30	0.001	0.043
ERG	-102.60	0.005	0.070
CAPZA2	-145.14	0.000	0.037
WDR61	-306.10	0.000	0.001

Table S7. Top common up- and down-regulated genes between human cirrhotic CD32b+ LSECs and CCl_4-cirrhotic rat LSECs.

Gene symbol	Fold-change		p-value		fdr	
	hLSECs	CCl_4	hLSECs	CCl_4	hLSECs	CCl_4
IGF2	10.69	3.95	0.000	0.000	0.037	0.000

TNFRSF4	5.34	5.31	0.012	0.000	0.098	0.000
DUSP2	4.62	2.40	0.001	0.022	0.046	0.037
TAGLN	3.95	5.57	0.045	0.000	0.174	0.000
NCF1	3.63	2.22	0.017	0.023	0.108	0.039
AEBP1	3.06	2.84	0.016	0.000	0.106	0.001
SLAMF8	2.87	2.82	0.011	0.001	0.093	0.004
EIF4EBP3	2.66	2.32	0.021	0.001	0.121	0.002
TACO1	-16.82	-2.06	0.032	0.020	0.148	0.034
METTL7A	-18.17	-2.04	0.005	0.000	0.071	0.001
ACO2	-18.36	-1.51	0.043	0.002	0.170	0.005
CABLES1	-18.68	-2.51	0.032	0.000	0.148	0.000
ATP5A1	-22.84	-1.61	0.021	0.003	0.120	0.008
VPS11	-23.35	-1.60	0.034	0.001	0.152	0.003
EXOC7	-25.60	-1.92	0.009	0.000	0.084	0.001
ICMT	-25.98	-2.16	0.008	0.000	0.083	0.000
ASAP3	-29.53	-2.09	0.001	0.000	0.046	0.001
XPNPEP1	-29.90	-1.65	0.014	0.001	0.102	0.004
DHDDS	-31.21	-2.21	0.014	0.000	0.101	0.000
LDHB	-32.10	-2.28	0.013	0.005	0.100	0.011
PGM5	-32.55	-1.51	0.033	0.002	0.149	0.006
CDK2AP1	-34.04	-1.50	0.009	0.001	0.084	0.004
UBE2G2	-41.71	-1.59	0.001	0.003	0.047	0.007
ALDH2	-49.32	-2.68	0.008	0.003	0.080	0.007
DCTN2	-61.63	-1.54	0.009	0.002	0.083	0.005

Table S8. Common DEG between cirrhotic human and CCl_4-cirrhotic rat LSECs defining extracellular exosomes components.

**Cellular Component: extracellular exosomes (GO:0070062)**	
**Gene symbol**	**Gene Name**
AEBP1 *	AE binding protein 1
AHCY	adenosylhomocysteinase
AKR1B1	aldo-keto reductase family 1 member B
ALDH2	aldehyde dehydrogenase 2 family (mitochondrial)
ANXA11	annexin A11
AP2M1	adaptor-related protein complex 2, mu 1 subunit
ARHGAP23	Rho GTPase activating protein 23
ARHGEF18	Rho/Rac guanine nucleotide exchange factor 18
*	BCR, RhoGEF and GTPase activating protein
BCR	calcium activated nucleotidase 1
CANT1	collagen type IV alpha 2 chain
COL4A2	copine 2
CPNE2	crystallin, lambda 1
CRYL1	dystroglycan 1
DAG1	dynactin subunit 2
DCTN2	damage-specific DNA binding protein 1
DDB1	dysferlin
DYSF	endothelin converting enzyme 1
ECE1	eukaryotic translation elongation factor 2

EEF2	eukaryotic translation initiation factor 5A
EIF5A	EPH receptor B4
EPHB4 *	endothelial cell adhesion molecule
ESAM	exostosin glycosyltransferase 2
EXT2	polypeptide N-acetylgalactosaminyltransferase 2
GALNT2	glucosylceramidase beta
GBA	galactosidase, beta 1
GLB1	guanine nucleotide binding protein, alpha 11
GNA11	glutamic-oxaloacetic transaminase 2
GOT2	glucose-6-phosphate isomerase
GPI	glutathione S-transferase kappa 1
GSTK1	hyaluronoglucosaminidase 1
HYAL1	hypoxia up-regulated 1
HYOU1	insulin-like growth factor 2
IGF2 *	junction plakoglobin
JUP	laminin subunit beta 2
LAMB2	LIM and SH3 protein 1
LASP1	lactate dehydrogenase B
LDHB	methyltransferase like 7A
METTL7A	multimerin 2
MMRN2	myosin 1C
MYO1C	nicastrin
NCSTN	Parkinsonism associated deglycase
PARK7	protocadherin 12
PCDH12	protocadherin gamma subfamily C, 3
PCDHGC3	phosphatidylethanolamine binding protein 1
PEBP1	prohibitin 2
PHB2	phospholipase C, delta 1
PLCD1	peptidylprolyl isomerase B
PPIB	polypyrimidine tract binding protein 1
PTBP1	pituitary tumor-transforming 1 interacting protein
PTTG1IP	RAB5B, member RAS oncogene family
RAB5B	RAB5C, member RAS oncogene family
RAB5C	similar to RIKEN cDNA 0610037L13
RGD155978	solute carrier family 25 member 25
6	solute carrier family 44 member 2
SLC25A25 *	serine and arginine rich splicing factor 2
SLC44A2	ST3 beta-galactoside alpha-2,3-sialyltransferase 4
SRSF2	stomatin
ST3GAL4	small ubiquitin-like modifier 3
STOM	transglutaminase 2
SUMO3	tubulointerstitial nephritis antigen-like 1
TGM2	target of myb1 like 2 membrane trafficking protein
TINAGL1 *	torsin family 1, member A
TOM1L2	torsin family 3, member A
TOR1A	tumor protein p63 regulated 1-like
TOR3A	tyrosylprotein sulfotransferase 2

TPRG1L	TNF receptor-associated protein 1
TPST2 *	tubulin, beta 6 class V
TRAP1	thioredoxin domain containing 5
TUBB6	ubiquitin-like modifier activating enzyme 1
TXNDC5	X-prolyl aminopeptidase 1
UBA1	
XPNPEP1	

* Denotes shared genes in 3 experiment models of CLD and human cells.

Table S9. Top common up- and down-regulated genes between human cirrhotic CD32b+ LSECs and TAA- cirrhotic rat LSECs.

Gene symbol	Fold-change		p-value		fdr	
	hLSECs	TAA	hLSECs	TAA	hLSECs	TAA
FN1	19.18	10.67	0.005	0.000	0.072	0.000
RARRES2	17.40	4.48	0.006	0.000	0.074	0.000
MXRA8	17.33	1.78	0.000	0.001	0.037	0.006
IGF2	10.69	3.60	0.000	0.000	0.037	0.001
NNMT	9.35	3.67	0.001	0.001	0.043	0.004
DACT3	9.29	4.84	0.000	0.000	0.013	0.001
LGALS1	7.56	8.94	0.000	0.000	0.037	0.000
AVPR2	7.28	1.88	0.005	0.005	0.070	0.014
SERPINF2	6.45	3.34	0.013	0.000	0.099	0.002
SPON2	6.02	3.69	0.002	0.000	0.057	0.001
HAS1	5.91	5.39	0.000	0.000	0.037	0.000
TNFRSF4	5.34	2.23	0.012	0.001	0.098	0.004
BOK	5.24	3.47	0.007	0.000	0.078	0.000
CLEC1B	-4.69	-2.13	0.015	0.000	0.104	0.000
LGMN	-4.70	-1.58	0.003	0.000	0.063	0.000
PLPP3	-5.15	-1.58	0.037	0.000	0.157	0.001
IRF2BP2	-5.60	-1.55	0.005	0.010	0.071	0.025
JADE2	-7.13	-1.84	0.007	0.000	0.076	0.001
GIMAP6	-9.97	-2.11	0.000	0.000	0.037	0.000
EHD3	-10.55	-1.99	0.001	0.001	0.043	0.003
TPST2	-12.15	-1.54	0.000	0.000	0.037	0.000
SLC31A2	-14.02	-2.38	0.034	0.000	0.152	0.000
TACO1	-16.82	-2.18	0.032	0.010	0.148	0.024
CABLES1	-18.68	-2.51	0.032	0.000	0.148	0.000
MID1	-18.89	-1.88	0.033	0.000	0.150	0.000

Table S10. Common DEG between cirrhotic human and TAA-cirrhotic rat LSECs defining extra-cellular exosomes components.

Cellular Component: extracellular exosomes (GO:0070062)	
**Gene symbol**	**Gene Name**
ADAM15	ADAM metallopeptidase domain 15
AEBP1 *	AE binding protein 1
APRT	adenine phosphoribosyl transferase
ARHGEF18 *	Rho/Rac guanine nucleotide exchange factor 18
CANT1	calcium activated nucleotidase 1
CLEC14A	C-type lectin domain family 14, member A

DDAH2	dimethylarginine dimethylaminohydrolase 2
EPHB4 *	EPH receptor B4
FLNA	filamin A
FN1	fibronectin 1
GALK1	galactokinase 1
IGF2 *	insulin-like growth factor 2
JADE2	jade family PHD finger 2
KRT18	keratin 18
LGALS1	galectin 1
LGMN	legumain
LTBP4	latent transforming growth factor beta binding protein 4
MAN1C1	mannosidase, alpha, class 1C, member 1
MXRA8	matrix remodeling associated 8
PAFAH1B3	platelet-activating factor acetylhydrolase 1b, catalytic subunit 3
PDLIM2	PDZ and LIM domain 2
PLPP3	phospholipid phosphatase 3
RARRES2	retinoic acid receptor responder 2
S100A6	S100 calcium binding protein A6
SERPINF2	serpin family F member 2
SLC1A5	solute carrier family 1 member 5
SLC25A25 *	solute carrier family 25 member 25
SLC7A8	solute carrier family 7 member 8
SNED1	sushi, nidogen and EGF-like domains 1
SPON2	spondin 2
TINAGL1 *	tubulointerstitial nephritis antigen-like 1
TPST2 *	tyrosylprotein sulfotransferase 2
VASN	vasorin
VWF	von Willebrand factor

* Denotes shared genes in 3 experiment models of CLD and human cells.

Table S11. Top common up- and down-regulated genes between human cirrhotic CD32b+ LSECs and cBDL- cirrhotic rat LSECs.

Gene symbol	Fold-change		p-value		fdr	
	hLSECs	cBDL	hLSECs	cBDL	hLSECs	cBDL
FN1	19.18	28.322	0.01	0.000	0.072	0.000
CFD	11.02	5.866	0.00	0.007	0.037	0.018
HK3	10.90	17.161	0.00	0.001	0.059	0.003
IGF2	10.69	2.018	0.00	0.027	0.037	0.052
CITED4	9.06	2.182	0.00	0.007	0.046	0.019
CORO1A	9.05	9.720	0.00	0.000	0.050	0.002
SLC16A3	8.63	11.936	0.00	0.000	0.013	0.000
LGALS1	7.56	30.311	0.00	0.000	0.037	0.000
SYTL1	7.40	1.815	0.00	0.004	0.061	0.011
HCST	7.32	4.756	0.00	0.000	0.041	0.001
S100A6	6.94	18.036	0.00	0.000	0.037	0.000
DOK2	6.84	4.501	0.00	0.001	0.037	0.003
LRRC25	6.53	11.372	0.01	0.001	0.079	0.003
S1PR4	6.48	7.078	0.01	0.000	0.082	0.001
PYCARD	6.11	5.607	0.00	0.001	0.045	0.004

TINAGL1	-2.23	-3.530	0.03	0.000	0.131	0.001
BACE2	-2.29	-2.159	0.01	0.002	0.080	0.008
GIMAP5	-2.30	-2.669	0.02	0.000	0.125	0.000
MTSS1L	-2.33	-2.140	0.01	0.028	0.078	0.054
TBXA2R	-2.34	-2.656	0.04	0.000	0.156	0.001
BCR	-2.34	-2.005	0.01	0.018	0.100	0.038
TNS2	-2.42	-2.618	0.01	0.000	0.076	0.001
MASP1	-40.57	-3.426	0.00	0.008	0.069	0.020
ADI1	-45.06	-2.032	0.01	0.025	0.078	0.049
ERG	-102.60	-1.876	0.00	0.007	0.070	0.019

Table S12. Common DEG between cirrhotic human and cBDL-cirrhotic rat LSECs defining extracellular exosomes components.

**Cellular Component: extracellular exosomes (GO:0070062)**	
**Gene symbol**	**Gene Name**
ADAM15	ADAM metallopeptidase domain 15
AEBP1 *	AE binding protein 1
ALDOA	aldolase, fructose-bisphosphate A
APRT	adenine phosphoribosyltransferase
ARHGAP23	Rho GTPase activating protein 23
ARHGEF18 *	Rho/Rac guanine nucleotide exchange factor 18
ARRDC1	arrestin domain containing 1
BCR	BCR, RhoGEF and GTPase activating protein
BEND7	BEN domain containing 7
C1QA	complement C1q A chain
CAPG	capping actin protein, gelsolin like
CD37	CD37 molecule
CFD	complement factor D
CLEC14A	C-type lectin domain family 14 member A
CORO1A	coronin 1A
CRYL1	crystallin lambda 1
CSK	c-src tyrosine kinase
CTSL	cathepsin L
DNPH1	2'-deoxynucleoside 5'-phosphate N-hydrolase 1
EPHB4 *	EPH receptor B4
FERMT3	fermitin family member 3
FGR	FGR proto-oncogene, Src family tyrosine kinase
FLNA	filamin A
FN1	fibronectin 1
GALK1	galactokinase 1
GRN	granulin precursor
GSTK1	glutathione S-transferase kappa 1
H2AFJ	H2A histone family member J
IGF2 *	insulin like growth factor 2
LGALS1	galectin 1
LSP1	lymphocyte-specific protein 1
MAN1C1	mannosidase alpha class 1C member 1
METRNL	meteorin like, glial cell differentiation regulator
MIF	macrophage migration inhibitory factor glycosylation-inhibiting factor
MMRN2	multimerin 2

MYO1G	myosin IG
NT5C	5', 3'-nucleotidase, cytosolic
NUDT1	nudix hydrolase 1
NUDT14	nudix hydrolase 14
PAFAH1B3	platelet activating factor acetylhydrolase 1b catalytic subunit 3
PCDH12	protocadherin 12
PDLIM2	PDZ and LIM domain 2
PGLS	6-phosphogluconolactonase
PLPP3	phospholipid phosphatase 3
PRDX5	peroxiredoxin 5
RASAL3	RAS protein activator like 3
RHOG	ras homolog family member G
RNH1	ribonuclease/angiogenin inhibitor 1
RPL37A	ribosomal protein L37a
RPLP1	ribosomal protein lateral stalk subunit P1
RPLP2	ribosomal protein lateral stalk subunit P2
RPS10	ribosomal protein S10
RPS14	ribosomal protein S14
RPS16	ribosomal protein S16
RPS19	ribosomal protein S19
RPS2	ribosomal protein S2
RPS28	ribosomal protein S28
RPS29	ribosomal protein S29
RPS3	ribosomal protein S3
RPS5	ribosomal protein S5
RPS9	ribosomal protein S9
S100A4	S100 calcium binding protein A4
S100A6	S100 calcium binding protein A6
SH3BGRL3	SH3 domain binding glutamate rich protein like 3
SLC1A5	solute carrier family 1 member 5
SLC25A25	solute carrier family 25 member 25
SLC2A1	solute carrier family 2 member 1
SNED1	sushi, nidogen and EGF like domains 1
ST14	suppression of tumorigenicity 14
ST6GAL1	ST6 beta-galactoside alpha-2,6-sialyltransferase 1
STOM	stomatin
SYNGR2	synaptogyrin 2
SYTL1	synaptotagmin like 1
TINAGL1 *	tubulointerstitial nephritis antigen like 1
TOM1L2	target of myb1 like 2 membrane trafficking protein
TPST2 *	tyrosylprotein sulfotransferase 2
TSPO	translocator protein
TWF2	twinfilin actin binding protein 2
VWF	von Willebrand factor

* Denotes shared genes in 3 experiment models of CLD and human cells.

Table S13. Top 25 up- & down-regulated genes in CCl_4-cirrhotic rat LSECs during CLD progression.

Gene symbol	Fold-change			p-value			fdr		
	Acute	Fibrosis	Cirrhosis	Acute	Fibrosis	Cirrhosis	Acute	Fibrosis	Cirrhosis

Ccl21	-8.75	-3.83	335.00	0.000	0.004	0.000	0.000	0.007	0.000
Clic5	-9.43	-3.39	26.83	0.000	0.007	0.000	0.000	0.011	0.000
Igsf3	23.98	20.65	25.93	0.000	0.000	0.000	0.000	0.000	0.000
Flnc	7.83	40.54	22.28	0.000	0.000	0.000	0.000	0.000	0.000
Mmp12	34.02	20.30	20.30	0.000	0.000	0.000	0.000	0.000	0.000
Cyp1a1	548.77	137.28	19.48	0.000	0.000	0.000	0.000	0.000	0.002
Lpl	9.55	3.12	19.44	0.002	0.003	0.000	0.004	0.004	0.000
Casc4	25.35	21.27	19.24	0.000	0.000	0.000	0.000	0.000	0.000
Il6	2.68	4.19	19.12	0.031	0.001	0.000	0.042	0.001	0.000
Prss23	6.06	12.33	17.82	0.000	0.000	0.000	0.000	0.000	0.000
Ednrb	-2.74	-1.52	15.82	0.000	0.036	0.000	0.000	0.049	0.000
Apba2	2.59	3.51	15.81	0.033	0.013	0.000	0.043	0.019	0.000
Plod2	5.43	22.54	15.26	0.000	0.000	0.000	0.000	0.000	0.000
Col12a1	4.62	22.32	14.73	0.001	0.000	0.000	0.001	0.000	0.000
Tll1	177.51	287.98	14.61	0.000	0.000	0.000	0.000	0.000	0.000
Cyp1b1	219.96	163.01	13.79	0.000	0.000	0.000	0.000	0.000	0.000
Dclk1	10.46	68.72	13.18	0.000	0.000	0.000	0.000	0.000	0.000
Abcc9	25.00	25.88	13.04	0.000	0.000	0.000	0.000	0.000	0.000
Lox	3.44	75.16	12.21	0.007	0.000	0.000	0.011	0.000	0.000
Il1rl1	3.19	14.57	8.85	0.019	0.000	0.000	0.026	0.000	0.000
Vash2	25.41	42.64	8.75	0.000	0.000	0.000	0.000	0.000	0.000
Igfbp5	-9.59	-2.61	8.58	0.000	0.002	0.000	0.000	0.004	0.000
Vegfd	2.42	3.59	8.12	0.008	0.000	0.000	0.012	0.000	0.000
Sulf1	3.48	14.99	7.96	0.001	0.000	0.000	0.002	0.000	0.000
Col14a1	-16.89	-3.36	7.86	0.000	0.002	0.000	0.000	0.004	0.000
Islr	-665.67	-262.01	-5.00	0.000	0.000	0.000	0.000	0.000	0.000
Gcat	-3.74	-5.53	-5.04	0.003	0.000	0.000	0.004	0.001	0.001
Dcxr	-48.04	-39.36	-5.10	0.000	0.000	0.000	0.000	0.000	0.002
Gpr153	-8.15	-10.47	-5.13	0.000	0.000	0.000	0.000	0.000	0.000
Akr7a2	-8.27	-10.71	-5.28	0.000	0.000	0.000	0.000	0.000	0.000
Hoxb5	-3.70	-8.03	-5.31	0.000	0.000	0.000	0.000	0.000	0.000
Cpne5	-31.19	-23.22	-5.33	0.000	0.000	0.000	0.000	0.000	0.000
Igdcc3	-3.26	-5.43	-5.33	0.000	0.000	0.001	0.001	0.000	0.004
Kcp	-31.78	-21.31	-5.43	0.000	0.000	0.000	0.000	0.000	0.000
Slco2b1	-169.94	-60.17	-5.48	0.000	0.000	0.000	0.000	0.000	0.000
Slc22a8	-65.84	-11.25	-5.67	0.000	0.001	0.006	0.000	0.002	0.013
Gpt	-14.68	-9.95	-6.35	0.000	0.000	0.001	0.000	0.001	0.003
Tmem132e	-10.29	-17.11	-6.61	0.000	0.000	0.000	0.000	0.000	0.000
Jph3	-21.00	-32.14	-6.62	0.000	0.000	0.000	0.000	0.000	0.000
Cib2	-8.61	-8.09	-6.93	0.000	0.000	0.000	0.000	0.000	0.000
Ptpru	-55.16	-75.61	-7.48	0.000	0.000	0.000	0.000	0.000	0.000
RGD13076 03	-378.74	-47.08	-7.81	0.000	0.000	0.003	0.000	0.000	0.007
Hal	-279.97	-65.99	-8.02	0.000	0.000	0.002	0.000	0.000	0.005
Cabp2	-365.01	-68.23	-8.22	0.000	0.000	0.001	0.000	0.000	0.003
Igf2bp2	10.48	6.81	-8.89	0.000	0.000	0.000	0.000	0.000	0.000
Hba1	-141.44	-16.15	-9.48	0.000	0.012	0.034	0.001	0.017	0.055
Cntfr	-19.40	-35.32	-9.54	0.000	0.000	0.000	0.000	0.000	0.000
Stac3	-97.79	-42.77	-16.10	0.000	0.000	0.001	0.000	0.000	0.003
Grik4	-3.48	-5.87	-18.73	0.000	0.000	0.000	0.001	0.000	0.000
Ihh	-60.27	-65.78	-31.75	0.000	0.000	0.000	0.000	0.000	0.000

Table S14. Genes related to molecular complexes and membrane receptors necessary for EVs biogenesis and exocytosis. (Table A relative to healthy, Table B relative to the previous CLD stage).

A)

Gene Symbol	Fold-change			p-value			fdr		
	Acute	Fibrosis	Cirrhosis	Acute	Fibrosis	Cirrhosis	Acute	Fibrosis	Cirrhosis
Vps28	-2.73	-3.18	-2.12	0.000	0.000	0.000	0.000	0.000	0.001
Mvb12a	-3.51	-3.98	-2.66	0.000	0.000	0.000	0.000	0.000	0.000
Mvb12b	-1.95	-2.46	-1.74	0.000	0.000	0.000	0.000	0.000	0.001
Ap2s1	-4.95	-5.67	-2.91	0.000	0.000	0.000	0.000	0.000	0.000
Flot2	-5.07	-6.37	-2.58	0.000	0.000	0.000	0.000	0.000	0.000
Cd9	-7.63	-4.31	2.02	0.000	0.000	0.020	0.000	0.000	0.035
Cd81	-9.56	-10.07	-2.18	0.000	0.000	0.000	0.000	0.000	0.000
Rab3a	-24.78	-8.24	-1.44	0.000	0.000	0.030	0.000	0.000	0.049
Sphk1	-96.52	-25.51	-2.10	0.000	0.000	0.060	0.000	0.000	0.089
S1pr3	-2.41	1.42	4.15	0.040	0.202	0.000	0.053	0.237	0.000
S1pr4	-59.61	-15.87	-2.36	0.000	0.000	0.057	0.000	0.000	0.086
Cers4	-2.54	-3.85	1.15	0.000	0.000	0.393	0.000	0.000	0.456
Tsg101	1.82	1.58	1.15	0.000	0.000	0.101	0.000	0.000	0.139
Vps36	4.16	3.49	2.62	0.000	0.000	0.000	0.000	0.000	0.001
Chmp4bl1	1.44	1.81	-1.01	0.016	0.000	0.945	0.023	0.000	0.954
Vps4a	1.93	1.63	1.03	0.000	0.001	0.752	0.000	0.002	0.791
Cers6	12.93	7.49	1.76	0.000	0.000	0.044	0.000	0.000	0.069

B)

Gene Symbol	Fold-change			p-value			fdr		
	Acute	Fibrosis	Cirrhosis	Acute	Fibrosis	Cirrhosis	Acute	Fibrosis	Cirrhosis
Vps28	-2.73	-1.16	1.50	0.000	0.051	0.000	0.000	0.132	0.000
Mvb12a	-3.51	-1.14	1.49	0.000	0.058	0.000	0.000	0.143	0.001
Mvb12b	-1.95	-1.26	1.41	0.000	0.006	0.000	0.000	0.033	0.000
Ap2s1	-4.95	-1.14	1.95	0.000	0.046	0.000	0.000	0.123	0.000
Flot2	-5.07	-1.26	2.47	0.000	0.005	0.000	0.000	0.032	0.000
Cd9	-7.63	1.77	8.69	0.000	0.028	0.000	0.000	0.088	0.000
Cd81	-9.56	-1.05	4.61	0.000	0.339	0.000	0.000	0.485	0.000
Rab3a	-24.78	3.01	5.72	0.000	0.021	0.000	0.000	0.072	0.000
Sphk1	-96.52	3.78	12.15	0.000	0.003	0.000	0.000	0.022	0.000
S1pr3	-2.41	3.41	2.93	0.040	0.024	0.002	0.053	0.079	0.002
S1pr4	-59.61	3.76	6.72	0.000	0.006	0.000	0.000	0.033	0.000
Cers4	-2.54	-1.52	4.42	0.000	0.001	0.000	0.000	0.010	0.000
Tsg101	1.82	-1.15	-1.37	0.000	0.004	0.000	0.000	0.028	0.000
Vps36	4.16	-1.19	-1.33	0.000	0.004	0.000	0.000	0.027	0.000
Chmp4bl1	1.44	1.26	-1.83	0.016	0.061	0.000	0.023	0.147	0.000
Vps4a	1.93	-1.19	-1.57	0.000	0.007	0.000	0.000	0.038	0.000
Cers6	12.93	-1.73	-4.25	0.000	0.000	0.000	0.000	0.004	0.000

Table S15. Top up- & down-regulated proteins in CCl_4-cirrhotic rat LSECs sEVs during CLD progression. (Table A relative to Healthy, Table B relative to the previous CLD stage).

A)

Gene symbol	Fold-change			p-value		
	Acute	Fibrosis	Cirrhosis	Acute	Fibrosis	Cirrhosis
TPM1	1.772	1.777	4.964	0.535	0.417	0.098
TPM2	1.362	-1.830	2.070	0.394	0.349	0.351
ANXA1	-1.935	-1.337	2.309	0.139	0.500	0.153
PURA	-5.044	-4.919	-1.619	0.238	0.219	0.646
C1QC	-4.489	-5.981	-2.344	0.050	0.077	0.241
PGRMC2	-2.576	-2.878	-2.158	0.243	0.365	0.235
TTC39C	-2.511	-3.536	-1.972	0.417	0.174	0.250
SSBP1	-33.94	-52.71	-30.51	0.001	0.000	0.001
MRPL14	1.742	2.044	2.447	0.484	0.301	0.257
IGF2	2.758	3.766	2.155	0.028	0.036	0.129
H1f0	2.100	3.067	2.169	0.406	0.121	0.228
HNRPA1	2.202	2.243	3.045	0.582	0.371	0.113
GDI2	2.148	2.650	3.546	0.299	0.195	0.124

B)

Gene symbol	Fold-change			p-value		
	Acute	Fibrosis	Cirrhosis	Acute	Fibrosis	Cirrhosis
TPM1	1.772	1.003	2.793	0.535	0.997	0.208
TPM2	1.362	-2.492	3.788	0.394	0.183	0.160
ANXA1	-1.935	1.447	3.087	0.139	0.352	0.071
PURA	-5.044	1.025	3.038	0.238	0.988	0.442
C1QC	-4.489	-1.332	2.552	0.050	0.741	0.301
PGRMC2	-2.576	-1.117	1.334	0.243	0.924	0.763
TTC39C	-2.511	-1.408	1.793	0.417	0.795	0.489
SSBP1	-33.94	-1.553	1.727	0.001	0.481	0.428
MRPL14	1.742	1.173	1.194	0.484	0.219	0.811
IGF2	2.758	1.366	-1.781	0.028	0.515	0.287
H1f0	2.100	1.460	-1.414	0.406	0.585	0.418
HNRPA1	2.202	1.019	1.358	0.582	0.991	0.709
GDI2	2.148	1.234	1.338	0.299	0.682	0.640

Table S16. Clinical characteristics.

Clinical parameters	Healthy	Cirrhosis
Age	65.8 ± 6	63 ± 4.2
Male	4 (80%)	4 (100%)
Ethanol etiology	-	4 (100%)
Child Pugh A/B/C	-	1/2/1
MELD	-	15.3 ± 2.7
Decompensated	-	4 (100%)
Ascites	-	4 (100%)
N	5	4

Table S17. Primary Antibodies.

Antigen	Manufacturer	Reference	Dilution
CD32B	Thermofisher	MA5-32601	1:1000
CD31	Abcam	ab32457	1:500
CD63	LifeSpan Bio	LS-188589	1:1000

CD81	BioRad	MCA 1846	1:1000
GRP78	LifeSpan Bio	LS-C209574	1:1000
COX IV	Cell Signaling	CS 11967	1:1000
TPM1	Cell Signaling	CS 3910	1:1000
α-SMA	Sigma	2547	1:200
α-Tubulin	Sigma	T9026	1:1000

Table S18. RT-qPCR primers.

Gene	Primer Sequence	Species
Gapdh	F:GGCATCGTGGAAGGGCTCAT<br>R:AGGGATGATGTTCTGGGCTGC	Rat
Col1α1	F:GTACATCAGCCCAAACCCCA<br>R:TCGCTTCCATACTCGAACTGG	Rat
Pdgfrβ	F:GTCAATGTCCCTGTCCGTGT<br>R:GTGTGGGTGACAGTTTTCGC	Rat
Hnf4α	F:ACTCTCTAAAACCCTCGCCG<br>R:TCAGATGGGGATGTGTCATTGC	Rat
Alb	F:TTTCGCCGAGAAGCACACAA<br>R:TCGGCATTCTCATCAGCGAC	Rat
Slc10a1	F:TCAAGCCTCCAAAGGACCAA<br>R:GCCATTAGGGGAAGGACCAG	Rat
Slc22a1	F:CAGACAGGTTTGGCCGTAAG<br>R:GTAGCCAGAGCCGACAAACT	Rat
Mrc1	F:TCAACTCTTGGACTCACGGC<br>R:CATGATCTGCGACTCCGACA	Rat
Il-10	F:CCTGGTAGAAGTGATGCCCC<br>R:AGACACCTTTGTCTTGGAGCTTAT	Rat
Il-6	F:TCCGGAGAGGAGACTTCACA<br>R:GCCATTGCACAACTCTTTTCT	Rat
Il-1β	F:GCTTCCTTGTGCAAGTGTCT<br>R:TCTGGACAGCCCAAGTCAAG	Rat
Acta2	F:CTCATGCCATCATGCGTCTG<br>R:CACGCTCAGCAGTAGTCACG	Rat

Supplementary Figures

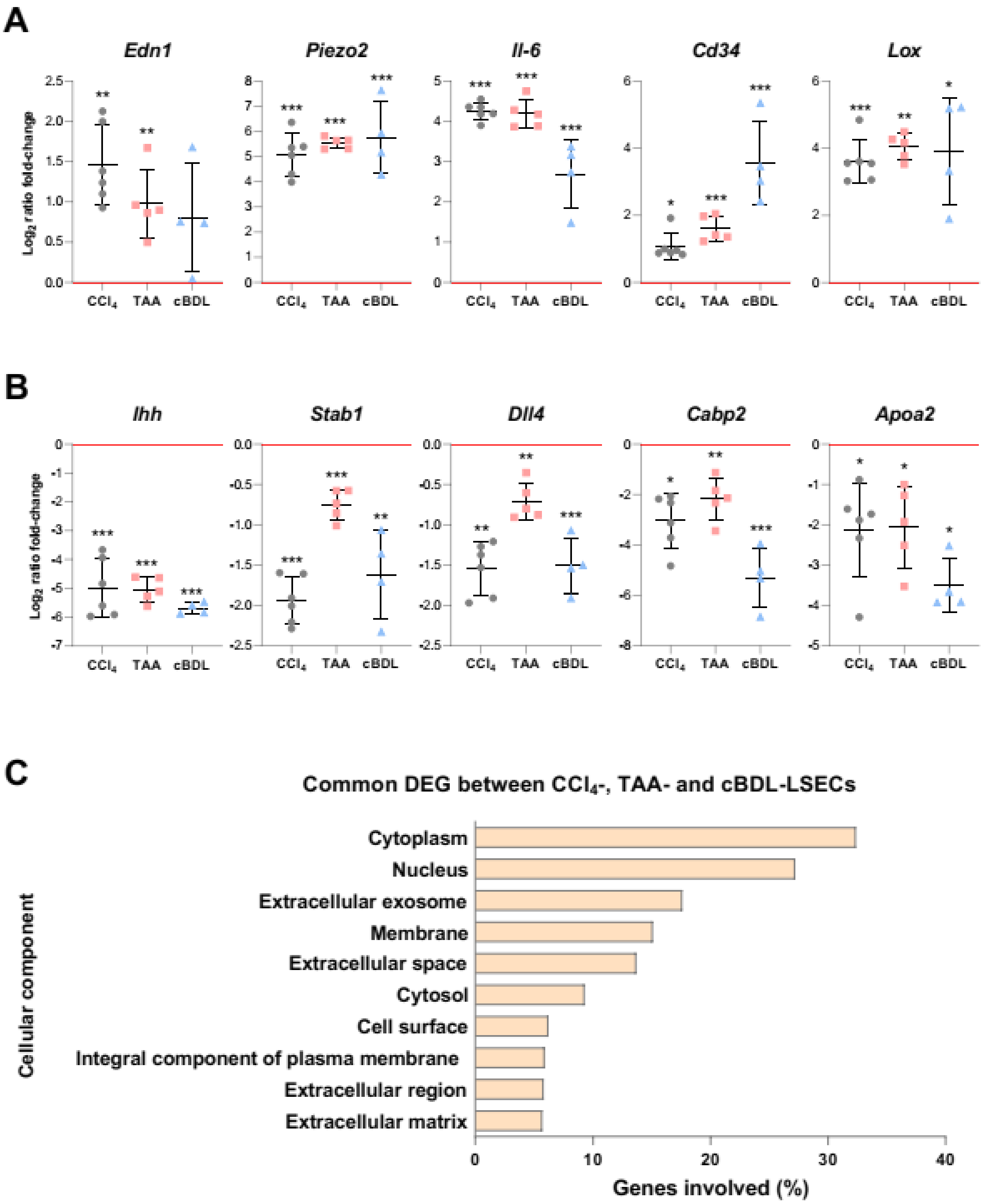

Figure S1. Common deregulated genes in CCl4-, TAA- and cBDL-cirrhotic LSECs. Up-regulated (A) and down-regulated (B) common characteristic genes in LSECs isolated from CCl4-, TAA- and cBDL-cirrhotic rats compared with corresponding healthy LSECs. Red line corresponds to healthy group. *p<0.05, **p<0.01 and ***p<0.001 vs. healthy cells. (C)Main cellular components described by Gene Ontology analyses of common differentially expressed genes (DEG) in three pre-clinical models of CLD. All cellular components are significant with p-value<0.05.

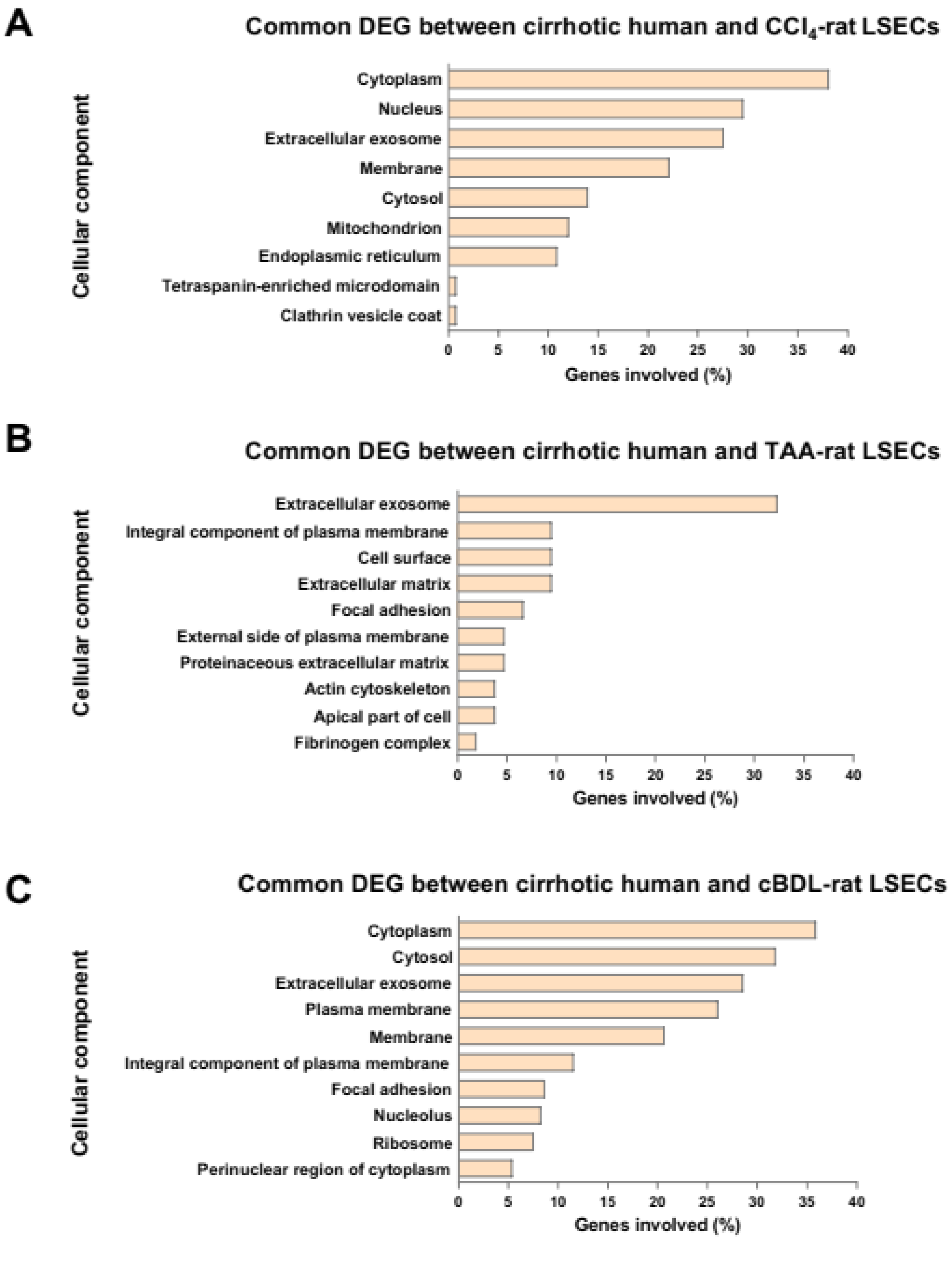

Figure S2. Cellular component analysis of commonly deregulated genes. Common deregulated genes analysed by Gene Ontology in LSECs from human cirrhotic and CCl4-LSECs (A), TAA-LSECs (B), and cBDL-LSECs (C). All cellular components are significant with p-value<0.05.

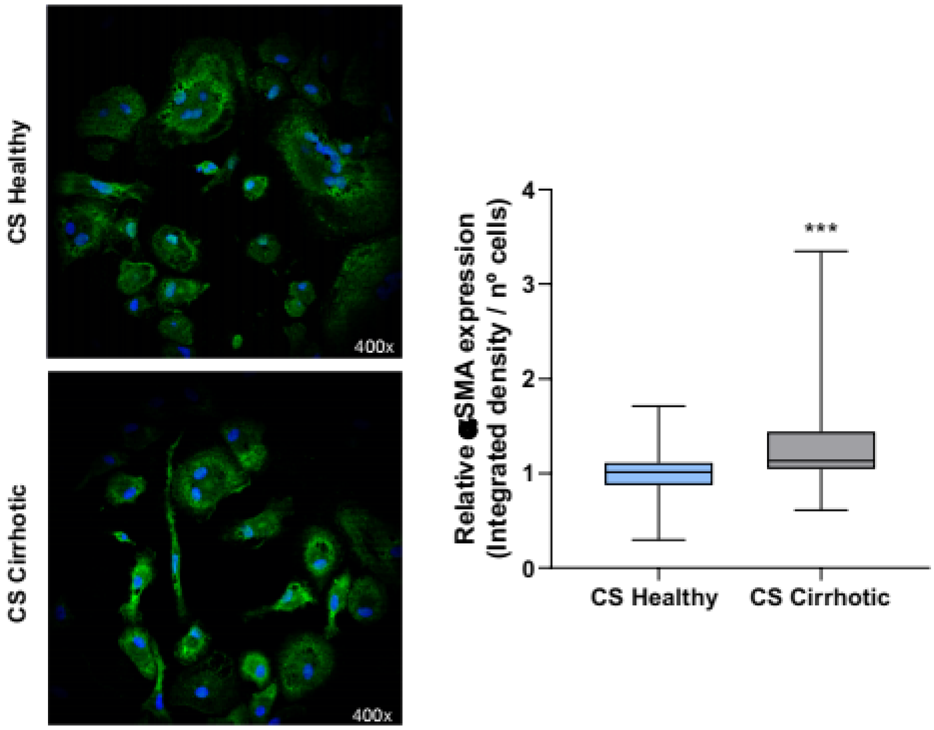

Figure S3. Effects of cirrhotic LSECs secretome in rat HSCs. Representative images of αSMA immunofluorescence (green, 400X magnification) in healthy rat HSCs treated for 48h with 550 ng/µL of complete secretome (CS) from healthy or cirrhotic rat LSECs, and corresponding quantification. ***p<0.001 (n=3 independent experiments) performing a t test with Mann-Whitney U test for non-parametric variables between groups.

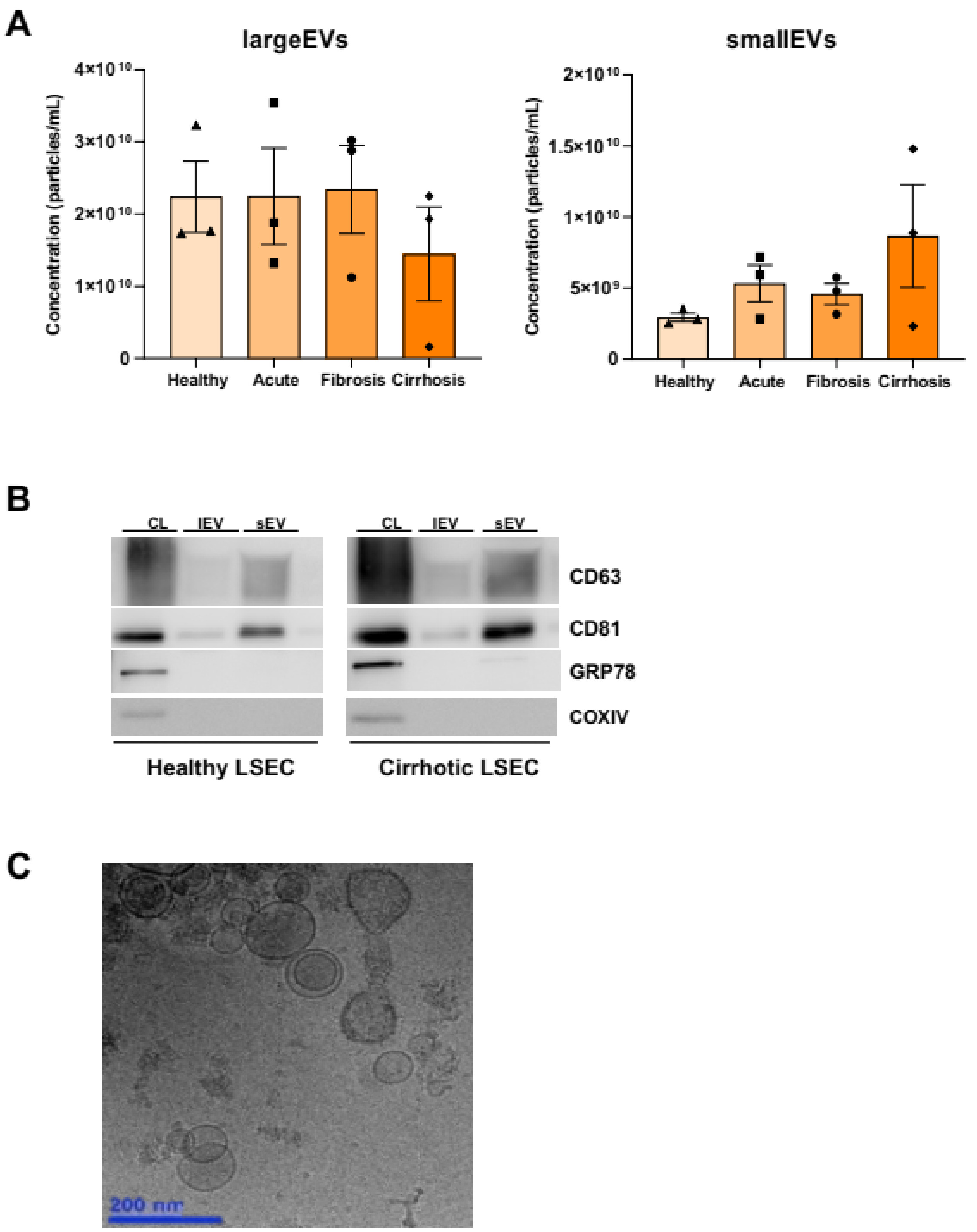

Figure S4. LSECs extracellular vesicles characterization. (A) Large and small extracellular vesicles (EVs) concentration in LSECs supernatant derived sub-fractions. (B) Protein expression of EVs markers in different secretome sub-fractions. (C) Representative image of EVs analysed by cryo-transmission electron microscopy. CL, cell lysate; lEVs, large extracellular vesicles, and sEVs, small extracellular vesicles. n=3 independent experiments.

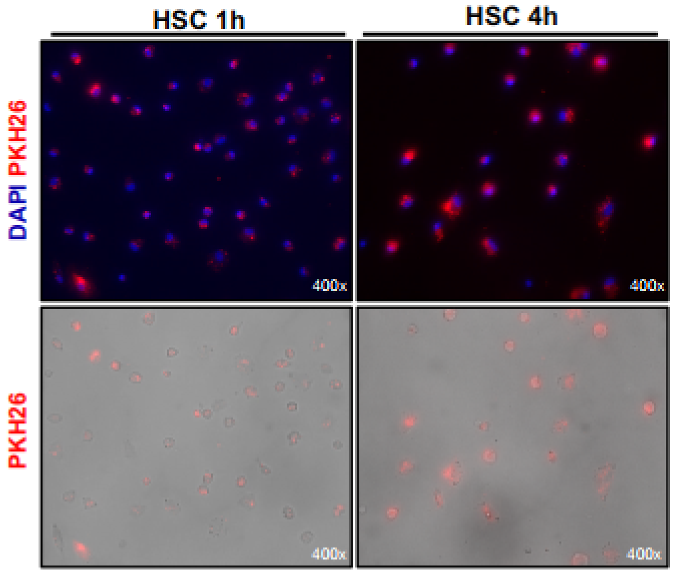

Figure S5. Extracellular vesicles internalization by HSCs. Small extracellular vesicles (sEVs) from cirrhotic LSECs were fluorescence-labelled and added to healthy rat HSCs at a concentration of 15 ng/μL. Representative images (400X magnification) shown after 1 and 4 hours of incubation. PKH26 (red), DAPI (blue).

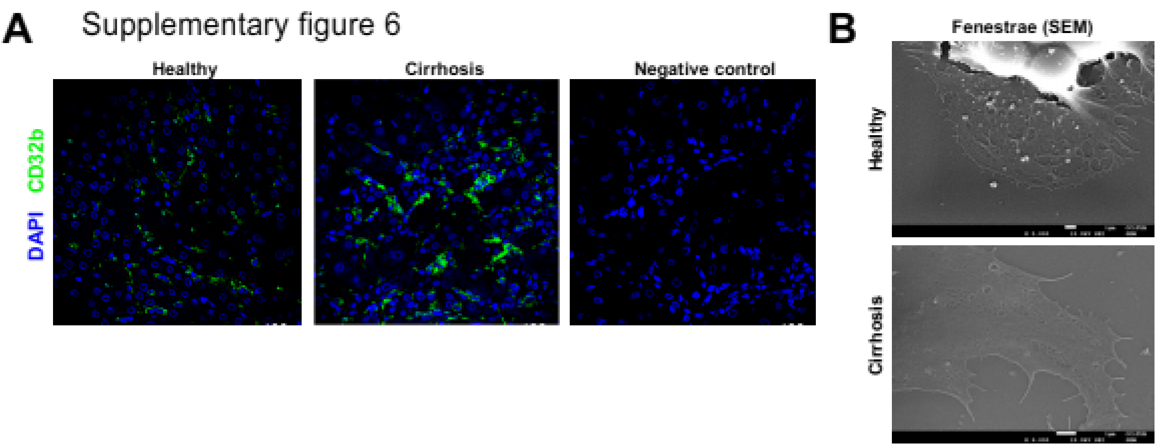

C

Description	setSize	Enrichment Score	NES	pvalue	p.adjust	qvalues	rank	leading_edge
SignatureD	381	-0.50908	-2.75954	1.62E-05	9.87E-05	2.83E-05	1595	tags=54%, list=31%, signal=40%
Endothelia1	71	-0.52958	-2.25232	1.79E-05	9.87E-05	2.83E-05	1526	tags=69%, list=29%, signal=50%
SignatureC	182	-0.34675	-1.72299	6.85E-05	0.000251	7.21E-05	1441	tags=36%, list=28%, signal=27%
Endothelia4	63	-0.45403	-1.88501	0.000325	0.000893	0.000256	1062	tags=40%, list=20%, signal=32%
SignatureB	620	-0.24094	-1.36425	0.001385	0.003047	0.000875	1073	tags=28%, list=21%, signal=26%
SignatureA	88	-0.35924	-1.58985	0.005281	0.009683	0.00278	1375	tags=43%, list=26%, signal=32%
Endothelia3	20	-0.54885	-1.73416	0.008748	0.013747	0.003947	1046	tags=65%, list=20%, signal=52%
Endothelia6	9	-0.62212	-1.55132	0.040951	0.056308	0.016165	1019	tags=56%, list=20%, signal=45%
Endothelia2	26	0.350598	1.222959	0.185101	0.212914	0.061124	742	tags=31%, list=14%, signal=27%
Endothelia5	36	-0.32838	-1.20529	0.193558	0.212914	0.061124	1138	tags=36%, list=22%, signal=28%
SignatureE	40	0.266743	1.034851	0.392981	0.392981	0.112817	1553	tags=45%, list=30%, signal=32%

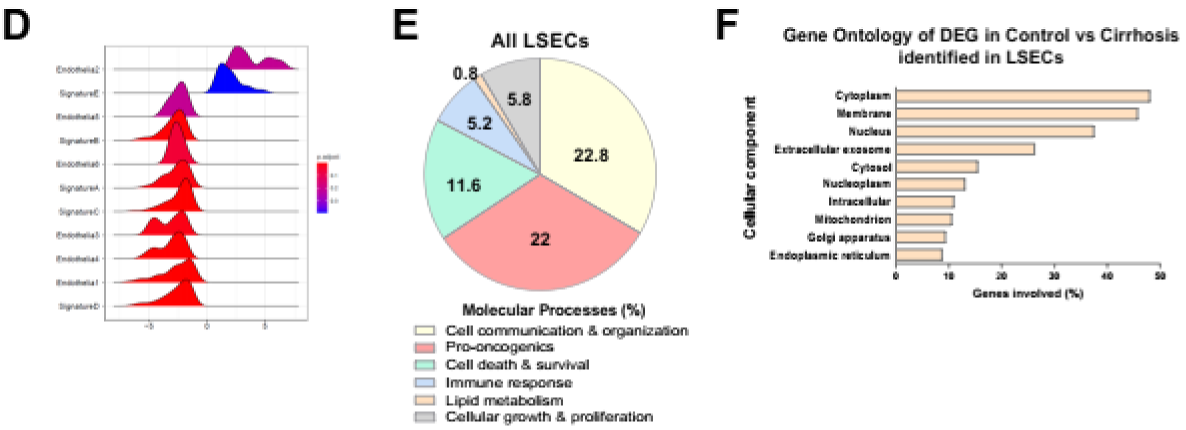

G

		RSEM expected counts (median ± SD)							
		Human		CCl_4		TAA		cBDL	
	Gene	Healthy	Cirrhosis	Healthy	Cirrhosis	Healthy	Cirrhosis	Healthy	Cirrhosis
LSECs	*Stab1*	833637 ± 250330.4	329958 ± 126056.8	135188 ± 22266.7	52990 ± 8512.9	70154 ± 9415.2	37783 ± 3413	68849 ± 6212.3	19663 ± 8532.9
	Vegfr3	51986 ± 11715.9	13429.5 ± 5314.2	30673.5 ± 6320.9	27147.5 ± 2976	32095 ± 6501.8	23844 ± 1546	31744 ± 2230.2	12058 ± 4818.3
HSCs	*Desmin*	0 ± 3.1	0 ± 0.0	261.5 ± 60.1	541 ± 143.7	43 ± 7.9	291 ± 153.2	46 ± 11.5	157 ± 479.5
	αSma	699 ± 534.9	247.5 ± 296.1	27 ± 18.3	342.5 ± 122.6	10 ± 9.2	650 ± 871	8 ± 4.4	230 ± 870.9
HMΦs	*Itgam*	858 ± 548.3	605.5 ± 516.3	935 ± 670.7	1537 ± 846.7	246 ± 101.9	85 ± 55.9	171 ± 126.2	4535 ± 6233.9
	Adgre1	21 ± 34	0 ± 42.5	1508.5 ± 770.8	2397 ± 1290.9	303 ± 170.3	136 ± 57.1	160 ± 169	2171 ± 1421.7
Hep	*Hnf4α*	4 ± 20.4	0 ± 0	3444 ± 3010.7	1177 ± 230.1	234 ± 87	277 ± 164.5	532 ± 623.2	167 ± 454
	Slc10a1	0 ±40.9	0 ± 0	895 ± 642.1	640 ± 376	23.3 ± 155	155 ± 69.2	330 ± 111.5	45 ± 50.5
	Slc22a1	36 ± 67.8	0.5 ± 45.3	638 ± 411.5	85.1 ± 31	16.7 ± 43	43 ± 13.5	115 ± 48.8	24 ± 10.1

Figure S6. Primary LSECs isolation. (A) Representative images of CD32B immunofluorescence (400X magnification) in healthy and cirrhotic (due to chronic alcohol consumption) human liver tissue. (B) Fenestrae visualization using scanning electron microscopy in healthy and cirrhotic human LSECs. (C) Results of Gene Set Enrichment Analysis (GSEA) comparing the human CD32b+ LSECs used in this study and different subsets of endothelial cells described in [12]. Human CD32b+ LSECs showed statistically significant high concordance with sub-population Endo(1) (identified as LSECs), Endo(3) and Endo(6) (scarassociated LSECs). Interestingly, 54% of

genes in our human cells were defined by the signature D, characterized by vesicle-mediated transport, blood vessel development, endocytosis and immune response as principal processes described by Gene Ontology. (D) Representative ridgeplot for expression distributions of enriched genes in different subpopulations and signatures. (E) Molecular processes and (F) Gene Ontology analyses of LSECs identified in single-cell sequencing described in [13]. All cellular components are significant with $p\text{-value} < 0.05$. (G) Cell isolation purity expressed as RSEM expected counts from RNAseq of marker genes of LSECs (Stab1 and Vegfr3), HSCs (αSma and Desmin), hepatic macrophages (Itgam and Adgre1) and hepatocytes (HNF4a, Slc10a1 and Slc22a1) determined in LSECs isolated from healthy and cirrhotic human and rat livers (median ± SD). SEM, scanning electron microscopy; NES, normalized enrichment scores; ns, not significant; CCl_4: CCl_4-induced rat cirrhosis; TAA: thioacetamide-induced rat cirrhosis; cBDL: common bile duct ligation-induced rat cirrhosis.

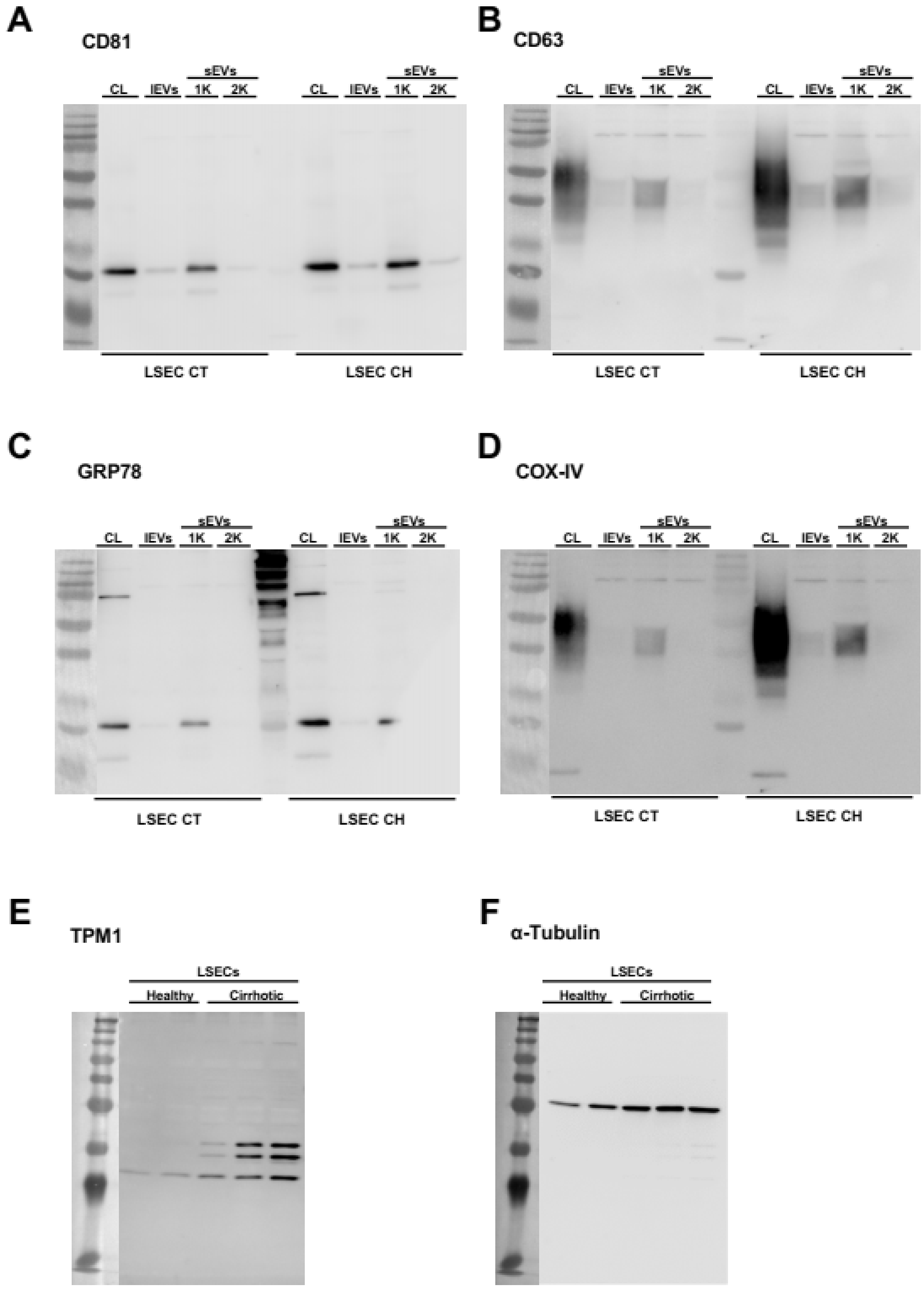

Figure S7. Western blot images. Whole nitrocellulose membranes of western blot assays performed in this study. (A) CD81, (B) CD63, (C) GRP78, (D) COXIV, (E) TPM1, and (F) α-Tubulin.

Supplementary References

1. Ortega-Ribera, M.; Fernández-Iglesias, A.; Illa, X.; Moya, A.; Molina, V.; Maeso-Díaz, R.; Fondevila, C.; Peralta, C.; Bosch, J.; Villa, R.; Gracia-Sancho, J. Resemblance of the Human Liver Sinusoid in a Fluidic Device With Biomedical And Pharmaceutical Applications. *Biotechnol Bioeng*. **2018**, *115* 2585–2594, doi:10.1002/bit.26776.

2. Øie, C.I.; Snapkov, I.; Elvevold, K.; Sveinbjørnsson, B.; Smedsrød, B. FITC Conjugation Markedly Enhances Hepatic Clearance of N-formyl Peptides. *PLoS One.* **2016**, 11, 160602, doi:10.1371/journal.pone.0160602.
3. Anania, J.C.; Chenoweth, A.M.; Wines, B.D.; Hogarth, P.M. The Human FcγRII (CD32) Family of Leukocyte FcR in Health and Disease. *Front. Immunol.* **2019**, *10*, 464, doi:10.3389/fimmu.2019.00464.
4. Ganesan, L.P.; Kim, J.; Wu, Y.; Mohanty, S.; Phillips, G.S.; Birmingham, D.J.; Robinson, J.M.; Anderson, C.L. FcγRIIb on Liver Sinusoidal Endothelium Clears Small Immune Complexes. *J. Immunol.* **2012**, *189*, 4981–4988, doi:10.4049/jimmunol.1202017.
5. Ramachandran, P.; Dobie, R.; Wilson-Kanamori, J.R.; Dora, E.F.; Henderson, B.E.P.; Luu, N.T.; Portman, J.R.; Matchett, K.P.; Brice, M.; Marwick, J.A.; et al. Resolving the fibrotic niche of human liver cirrhosis at single-cell level. *Nat. Cell Biol.* **2019**, *575*, 512–518, doi:10.1038/s41586-019-1631-3.
6. Théry, C.; Witwer, K.W.; Aikawa, E.; Alcaraz, M.J.; Anderson, J.D.; Andriantsitohaina, R.; Antoniou, A.; Arab, T.; Archer, F.; Atkin-Smith, G.K.; et al. Minimal information for studies of extracellular vesicles 2018 (MISEV2018): A position statement of the International Society for Extracellular Vesicles and update of the MISEV2014 guidelines. *J. Extracell. Vesicles* **2018**, *7*, 1535750, doi:10.1080/20013078.2018.1535750.
7. Wiśniewski, J.R.; Zougman, A.; Nagaraj, N.; Mann, M. Universal Sample Preparation Method for Proteome Analysis. *Nat Methods.* **2009**, *6*, 359–362, doi:10.1038/nmeth.1322.
8. Meier, F.; Beck, S.; Grassl, N.; Lubeck, M.; Park, M.A.; Raether, O.; Mann, M. Parallel Accumulation–Serial Fragmentation (PASEF): Multiplying Sequencing Speed and Sensitivity by Synchronized Scans in a Trapped Ion Mobility Device. *J. Proteome Res.* **2015**, *14*, 5378–5387, doi:10.1021/acs.jproteome.5b00932.
9. Meier, F.; Brunner, A.-D.; Koch, S.; Koch, H.; Lubeck, M.; Krause, M.; Goedecke, N.; Decker, J.; Kosinski, T.; Park, M.A.; et al. Online Parallel Accumulation–Serial Fragmentation (PASEF) with a Novel Trapped Ion Mobility Mass Spectrometer. *Mol. Cell. Proteom.* **2018**, *17*, 2534–2545, doi:10.1074/mcp.tir118.000900.
10. Schindelin, J.; Arganda-Carreras, I.; Frise, E; Kaynig, V.; Longair, M.; Pietzsch, T.; Preibisch, S.; Rueden, C.; Saalfeld, S.; Schmid, B.; et al. Fiji: An Open-Source Platform for Biological-Image Analysis. *Nature Methods.* **2012**, *9*, 676–682, doi:10.1038/nmeth.2019.
11. Su, T.; Yang, Y.; Lai, S.; Jeong, J.; Jung, Y.; McConnell, M.; Utsumi, T.; Iwakiri, Y. Single-Cell Transcriptomics Reveals Zone-Specific Alterations of Liver Sinusoidal Endothelial Cells in Cirrhosis. *Cell. Mol. Gastroenterol. Hepatol.* **2021**, *11*, 1139–1161, doi:10.1016/j.jcmgh.2020.12.007.
12. Ramachandran, P.; Dobie, R.; Wilson-Kanamori, J.R.; Dora, E.F.; Henderson, B.E.P.; Luu, N.T.; Portman, J.R.; Matchett, K.P.; Brice, M.; Marwick, J.A.; et al. Resolving the fibrotic niche of human liver cirrhosis at single-cell level. *Nat. Cell Biol.* **2019**, *575*, 512–518, doi:10.1038/s41586-019-1631-3.
13. Su, T.; Yang, Y.; Lai, S.; Jeong, J.; Jung, Y.; McConnell, M.; Utsumi, T.; Iwakiri, Y. Single-Cell Transcriptomics Reveals Zone-Specific Alterations of Liver Sinusoidal Endothelial Cells in Cirrhosis. *Cell. Mol. Gastroenterol. Hepatol.* **2021**, *11*, 1139–1161, doi:10.1016/j.jcmgh.2020.12.007.
